# Expert-based medication reviews to reduce polypharmacy in older patients in primary care: a northern-Italian cluster-randomised controlled trial

**DOI:** 10.1186/s12877-021-02612-0

**Published:** 2021-11-23

**Authors:** Angelika Mahlknecht, Christian J. Wiedermann, Marco Sandri, Adolf Engl, Martina Valentini, Anna Vögele, Sara Schmid, Felix Deflorian, Carmelo Montalbano, Dara Koper, Romuald Bellmann, Andreas Sönnichsen, Giuliano Piccoliori

**Affiliations:** 1 Institute of General Practice and Public Health, College of Health Care Professions, Lorenz Böhler- Street 13, 39100 Bolzano, Italy; 2 Institute of General Practice, Family Medicine and Preventive Medicine, Paracelsus Medical University, Strubergasse 21, 5020 Salzburg, Austria; 3 UMIT – Private University for Health Sciences, Medical Informatics and Technology – Tyrol, Eduard-Wallnöfer-Zentrum 1, 6060 Hall in Tirol, Austria; 4 Big & Open Data Innovation Laboratory (BODaI-Lab), University of Brescia, Via S. Faustino 74/B, 25122 Brescia, Italy; 5 South Tyrolean Academy of General Practice, Wangergasse 18, 39100 Bolzano, Italy; 6 Genomedics S.r.L. Health Care Consultants, Via Sestese 61, 50141 Florence, Italy; 7 Salzburger Gesundheitsfonds, Sebastian Stief-Gasse 2, 5020 Salzburg, Austria; 8 Clinical Pharmacokinetics Unit, Division of Medical Intensive Care and Emergency Medicine, Department of Internal Medicine I, Medical University of Innsbruck, Peter-Anich- Street 35, 6020 Innsbruck, Austria; 9 Department of General Practice and Family Medicine, Center for Public Health, Medical University of Vienna, Kinderspitalgasse 15/I, 1090 Vienna, Austria

**Keywords:** Polypharmacy, Older adults, General practice, Medication review, Inappropriate prescribing

## Abstract

**Background:**

Evidence regarding clinically relevant effects of interventions aiming at reducing polypharmacy is weak, especially for the primary care setting. This study was initiated with the objective to achieve clinical benefits for older patients (aged 75+) by means of evidence-based reduction of polypharmacy (defined as ≥8 prescribed drugs) and inappropriate prescribing in general practice.

**Methods:**

The cluster-randomised controlled trial involved general practitioners and patients in a northern-Italian region. The intervention consisted of a review of patient’s medication regimens by three experts who gave specific recommendations for drug discontinuation.

Main outcome measures were non-elective hospital admissions or death within 24 months (composite primary endpoint). Secondary outcomes were drug numbers, hospital admissions, mortality, falls, fractures, quality of life, affective status, cognitive function.

**Results:**

Twenty-two GPs/307 patients participated in the intervention group, 21 GPs/272 patients in the control group. One hundred twenty-five patients (40.7%) experienced the primary outcome in the intervention group, 87 patients (32.0%) in the control group. The adjusted rates of occurrence of the primary outcome did not differ significantly between the study groups (intention-to-treat analysis: adjusted odds ratio 1.46, 95%CI 0.99–2.18, *p* = 0.06; per-protocol analysis: adjusted OR 1.33, 95%CI 0.87–2.04, *p* = 0.2).

Hospitalisations as single endpoint occurred more frequently in the intervention group according to the unadjusted analysis (OR 1.61, 95%CI 1.03–2.51, *p* = 0.04) but not in the adjusted analysis (OR 1.39, 95%CI 0.95–2.03, *p* = 0.09). Falls occurred less frequently in the intervention group (adjusted OR 0.55, 95%CI 0.31–0.98; p = 0.04). No significant differences were found regarding the other outcomes.

Definitive discontinuation was obtained for 67 (16.0%) of 419 drugs rated as inappropriate.

About 6% of the prescribed drugs were PIMs.

**Conclusions:**

No conclusive effects were found regarding mortality and non-elective hospitalisations as composite respectively single endpoints. Falls were significantly reduced in the intervention group, although definitive discontinuation was achieved for only one out of six inappropriate drugs. These results indicate that (1) even a modest reduction of inappropriate medications may entail positive clinical effects, and that (2) focusing on evidence-based new drug prescriptions and prevention of polypharmacy may be more effective than deprescribing.

**Trial registration:**

Current Controlled Trials (ID ISRCTN: 38449870), date: 11/09/2013.

**Supplementary Information:**

The online version contains supplementary material available at 10.1186/s12877-021-02612-0.

## Background

Prescription and monitoring of drug therapy in older patients is challenging due to age-related physiological changes and frequent concomitant conditions [1]. Moreover, evidence regarding drug therapy in older adults is scarce or shows major research deficits: guidelines are often based on expert consensus or do not consider common comorbidities, e.g. cognitive dysfunction [2]. Benefits of therapies are usually demonstrated by trials involving younger and healthier persons and results might not be applicable to older-aged multimorbid patients [3] whose life expectancies are sometimes shorter than the time required to gain benefits from pharmacological treatments [4]. Clinical guidelines usually address single diseases and often require the use of several drugs per disease. Moreover, following new guidelines with lower thresholds for starting pharmacological treatment and the presence of new medications may induce physicians to prescribe more drugs [5]. Thus, in the treatment of patients with multiple chronic conditions, guideline adherence (as demanded e.g. by quality programmes) inevitably leads to the use of multiple medications [6].

As a consequence, polypharmacy has become a major health concern in older adults [7–9] and has been well documented to entail an increased risk of potentially inappropriate medication (PIM) [10, 11], adverse drug events (ADEs) [12], medication non-adherence [13] and increased healthcare costs [14]. Polypharmacy was also demonstrated to be an independent predictor of nursing home admission, malnutrition, fractures, impaired mobility [15] and to be associated with higher rates of preventable hospitalisations [16, 17], hospital re-admissions within short periods [18] and increased mortality [19].

In primary care, studies showed up to 40% of patients aged ≥65 years [20] respectively 54% of patients aged ≥70 years [21] to be affected from polypharmacy. In the long-term inpatient setting, the prevalence of polypharmacy is even higher [22].

Thus, the evidence clearly indicates that there is a strong need to reduce polypharmacy and inappropriate prescribing. Several strategies in this regard have been investigated in various settings: medication reviews performed by pharmacists and/or employing other healthcare professionals [23], multidisciplinary case conferences [24], pharmacist consultations [25], educational programmes [26], computerised support systems and multifaceted approaches [25]. The interventions seemed to be effective in reducing inappropriate prescribing, however, the overall quality of the evidence was considered to be low and convincing effects on clinically relevant outcomes such as hospitalisations or mortality could not be demonstrated [23].

Nevertheless, given the high prevalence of polypharmacy and its potentially harmful impact on health outcomes, it seems plausible that reducing polypharmacy will influence clinical outcomes favourably. In the geriatric inpatient setting, a promising strategy has been investigated by Garfinkel et al. which consisted of drug regimens’ evaluation and discontinuation, change or dose reduction of medications by using an ad hoc-developed algorithm. The study showed a reduction of mortality and acute hospitalisations, however, the study design was non-randomised and the sample was small [4]. Another five-step process to reduce polypharmacy (‘deprescribing’) seemed to be beneficial [27], but evidence is inconsistent [28].

As medical care of chronically ill patients is mainly assured by general practitioners (GPs) [29] and GPs assume a crucial role in prescription and monitoring of drug therapies [30], well-designed interventions to reduce polypharmacy and inappropriate prescribing are strongly required for general practice.

Therefore, the present cluster-randomised controlled trial (RCT) ‘PRIMA’ (**P**olypharmacy in chronic diseases- **R**eduction of **I**nappropriate **M**edication and **A**dverse drug events in older populations) [31] was initiated with the aim to study if the prudent, evidence-based intervention to reduce polypharmacy leads to benefits for multimorbid older patients in the primary care setting.

The intervention comprised an appraisal of each patient’s medication regimen by three experts who gave specific recommendations for drug discontinuation to support the GPs in reducing polypharmacy and inappropriate drug use.^1^

## Methods

The CONSORT guidelines for randomised trials of the EQUATOR network (**E**nhancing the **QUA**lity and **T**ransparency **O**f health **R**esearch) were followed in the preparation of the study report.

### Study design, population and setting

The cluster-RCT (12/2012–11/2016) was conducted in the primary care setting and involved GPs and older-aged community-living patients in the province of Bolzano (Italy). The observation period was planned to be 24 months.

### Calculation of sample size

The calculation was based on the results of the above-mentioned study by Garfinkel et al. [4] who used a geriatric-palliative algorithm for prudent reduction of polypharmacy and established the annual rate of acute hospitalisation as primary endpoint. The study observed an absolute risk reduction of 18% regarding the primary endpoint (corresponding relative risk reduction: 60%).

As the effects in non-randomised trials are usually overestimated, the setting of the Garfinkel study was a geriatric clinic and our primary endpoint was different (non-elective hospital admission or death within 24 months), we presumed to achieve a relative risk reduction of 35–40%. We expected to recruit healthier patients and therefore estimated an annual rate of 60% of the rate observed by Garfinkel thus achieving an estimated annual rate of hospitalisations/death of 12.5% for the control group, corresponding to 25% in two years. For the intervention group, a relative risk reduction of 37% was expected (absolute risk reduction 9.3%) corresponding to an event rate of 15.8% in two years.

Assuming α = 0.05 and a power of 1-β = 0.8, we calculated a sample size of 602 patients (301 per arm). After considering a drop-out rate of 10%, the necessary sample size was 666 patients (333 per arm). Assuming a recruitment of 10–15 patients per GP office we aimed at recruiting 44–67 GPs.

As less GPs were recruited than a priori expected (43 GPs), the sample size was re-calculated assuming a larger risk reduction from 25 to 15% instead of 15.8%, achieving thus a final necessary sample size of 543 patients (272 per arm, 13 patients per GP).

### Recruitment of study participants and instruction of GPs

#### Recruitment of GPs

Recruitment was conducted between 01/2014 and 10/2014. The research team informed and invited to participate all 270 active GPs listed in the local Chamber of Physicians by email and phone.

The participating physicians were informed about the study in an initial onsite meeting and were instructed regarding the collection of data, the electronic generation of the case report forms (CRFs, see below) and the procedure of the intervention. The GPs also received a written handout and video tutorials as instruction. Moreover, throughout the whole study period, the GPs were personally supported by the research team regarding all study procedures by means of telephonic contacts and personal visits in the GP offices.

#### Recruitment of patients

The participating GPs identified consecutive eligible patients and invited them to participate during routine visits in the GP office.

All participating GPs and patients gave written informed consent. The GPs were remunerated for participation.

### Inclusion criteria (patients)

- Age ≥ 75 years
- On therapy with ≥8 prescribed active agents. The cut-off of ≥8 drugs was chosen as the development of our study concept and sample size calculation was based on the results of a former study realised by Garfinkel et al. [4] (see above) which used a geriatric-palliative algorithm as deprescribing intervention and found a consumption of > 7 drugs among the included older-aged participants. This was the basis for the cut-off chosen for our study by defining as inclusion-criterion the corresponding number of > 7 drugs, i.e. ≥8 drugs.
- Combination drugs were counted according to the number of active agents. PRN-medications and OTC-drugs were excluded as the analysis was limited to *chronic* therapies and to *prescribed* medications which were the only electronically extractable drugs from the GPs’ electronic health records (EHRs) during data collection.

### Exclusion criteria (patients)

Life expectancy less than three months according to the GP’s judgement, advanced stage of cancer, radiation/chemotherapy, severe cognitive impairment with inability to give informed consent.

### Cluster-randomisation

The participating GPs were randomised by the project team using computerised sequence generation. To avoid contamination risks, units of randomisation were the GPs; thus, all included patients of one participating GP were either participating to the intervention group (IG) or to the control group (CG). The participating GPs were stratified according to gender and location (urban or rural area) to avoid over-representation of a specific feature in one group.

At the time of baseline data collection, neither GPs nor patients knew if they would be part of the intervention or control group (allocation concealment).

Blinding of participating GPs and patients was not possible due to the nature of the intervention.

### Intervention

#### Pre-review

The medication plans of all participating patients, also of the control group, were pre-reviewed by a GP of the project team who was not included as study participant and by a student of pharmacology. They checked the medication plans for *PIMs* using the 2012 Beers criteria (Italian Version) [32, 33] and for drug-drug interactions (*DDIs*) using the Lexicomp/UpToDate® database [34]; only potentially severe DDIs were considered (categories D = consider drug modification and X = avoid combination).

The pre-review was carried out (a) to describe the *whole study sample* in terms of prevalence of PIMs, DDIs and associated factors [31], and (b) for the *intervention group* to provide the experts with information serving as a basis for the elaboration of the deprescribing recommendations (see below). The results of the pre-review were not communicated to the participating GPs at any time.

#### Intervention

Subsequently, every drug regimen was assessed by a specialist in internal medicine, a clinical pharmacologist, and an evidence-based medicine (EBM) expert in due consideration of current best evidence regarding pharmacological treatment in older patients, of disease-specific guidelines, and by considering the results of the pre-review, i.e. presence of PIM and/or severe DDIs. The experts were given all patient information documented on the CRFs (see below). As it was the scope of the intervention that each expert conducted the assessments using his profession-specific expertise, the project team was not informed in detail regarding additional instruments used by the experts (e.g., electronic decision aids).

Based on their medication review, the experts elaborated recommendations for discontinuation of inappropriate drugs and sent them to the research team. There was no prioritisation of drugs to be discontinued.

If at least two experts concorded regarding a specific recommendation, the respective recommendation and a brief explanation was forwarded to the respective GP by the research team. The GPs were invited to reflect on the recommendations in a shared decision-making process with the patient. All final decisions regarding continuation/discontinuation of drugs remained in the responsibility of the GP and the patient. The GPs informed the project team if they adopted the recommendations and gave an explanation statement in case of non-adherence. After three respectively six months, the research team contacted the GPs to recall the recommendations received and to discuss any difficulties regarding application.

**Fig. 1:**
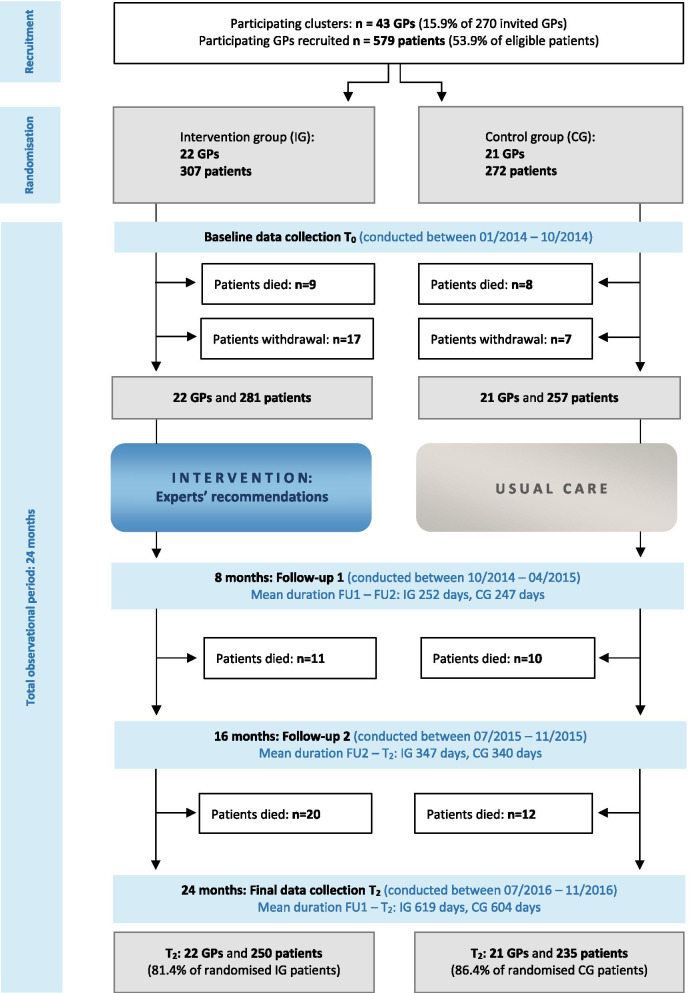
Flow diagram of GP and patient participation. GPs general practitioners, IG Intervention group, IG Control group, FU Follow up

The intervention took place between baseline data collection (T_0_) and the first follow-up (FU1) (Fig.1).

### Control

The patients in the control group received usual care. This included potential use of guidelines by their GPs and drug changes during routine care, but they did not receive the structured medication review with recommendations by the three experts.

The GPs in the control group recorded data of their included patients concordantly with the intervention group.

### Outcomes

The primary endpoint was a composite binary outcome of all-cause mortality or unplanned hospitalisations. This was chosen to allow that all patients could be included in the analysis, as the two main outcomes may compete. Additionally, all-cause mortality and non-elective hospitalisations were analysed as single secondary outcomes. All analysed primary and secondary endpoints and the respective measuring methods are described in Table 1.

**Table 1:** Primary and secondary outcomes

Composite primary outcome		
***Outcome***	***Measuring method***	***Description***
**Non-elective hospitalisations or all-cause mortality**	Recorded by the GP in the case report form (CRF) at FU1, FU2, T_2_ (final data analysis)	Hospitalisations: number of episodes (referral to any acute care facility, either emergency department or hospital; elicited by the GP, by any other physician or by the patient himself) Death: number of patients
**Secondary outcomes**		
***Outcome***	***Measuring method***	***Description***
**All-cause mortality**	All events were recorded by the GPs in the CRF at T_0_, FU1, FU2, T_2_	Number of patients
**Non-elective hospital admissions**	All events were recorded by the GPs in the CRF at T_0_, FU1, FU2, T_2_	Number of episodes (see primary endpoint)
**Falls**	All events were recorded by the GPs in the CRF at T_0_, FU1, FU2, T_2_	Number of falls requiring medical care
**Fractures**	All events were recorded by the GPs in the CRF at T_0_, FU1, FU2, T_2_	Number of fractures occurred within the observation period
**Number of drug prescriptions**	Recorded by the GPs in the CRF at T_0_, FU1, FU2, T_2_	Total number of prescribed drugs
**Health-related quality of life**	**EQ-5D-5L** and **EQ-VAS** [35] The questionnaire was handed out to the patients by the GPs at T_0_ and T_2_ (prior to / after the intervention)	- Five items addressing health-related QoL (mobility, self-care, usual activities, anxiety / depression, pain) and resulting in an index value (maximum = 1 = full health) - Visual analogue scale EQ-VAS (range 0–100)
**Affective status**	**5-Item Geriatric Depression Scale** (5-GDS) [36] The questionnaire was handed out to the patients by GPs at T_0_ and T_2_	- Five items addressing satisfaction, tediousness, helplessness, social withdrawal, self-esteem - ≥2 points → presence of depressive symptoms (range 0–5)
**Cognitive performance**	**6-Item Cognitive Impairment Test** (6-CIT) [37] The questionnaire was handed out to the patients by GPs at T_0_ and T_2_	- Six items evaluating cognitive function - ≥10 points → significant cognitive impairment (range 0–28)

*GPs* General practitioners, *CRF* Case Report Form, *T*_*0*_ Baseline data collection, *FU* Follow-up study visit, *T*_*2*_ Final study visit, *EQ-5D* 5-Item questionnaire measuring health-related quality of life, *VAS* Visual analogue scale, *QoL* Quality of life.

### Data collection

After obtaining informed consent, every participating patient was documented via a structured CRF. The CRF was provided in electronic form for those GPs who used the EHR Millewin® (*n* = 39) which allowed the electronic integration of the CRF. For this purpose, an add-on module was programmed which filled in automatically all required patient data available in the EHR. The GPs checked these electronically generated CRFs and completed missing data manually (e.g., the results of the patient questionnaires). The electronic CRFs were then sent to the research team as an email attachment via the add-on module.

The add-on module supported the GPs also during the recruitment and intervention period. Every time the GP opened a patient health record, e.g. during visits in the GP office, consultations by phone or in case of drug prescriptions, alerts and reminders popped up regarding eligibility of a patient (recruitment phase) or regarding missing actions/documentations (intervention phase).

For the remaining *n* = 4 GPs using other EHRs, the CRFs were provided on paper. The GPs completed the paper CRFs manually and sent them via fax or email to the research group.

The GPs of both the intervention and the control group recorded at *baseline* (T_0_, prior to the intervention) the following parameters in the CRF:

- Patients’ age, sex, diagnoses (ICD-9-coded), current drug prescriptions (international non-proprietary names, ATC-coded), daily dosage of the prescribed drugs
- Biometric and laboratory parameters: height, weight, BMI, blood pressure, renal functional parameters, potassium, cardiac frequency, haemoglobin, HbA1c, hepatic enzymes, erythrocyte sedimentation rate, brain natriuretic peptide (BNP/NT-proBNP), International Normalised Ratio (INR)^2^
- Symptoms: Nausea, vertigo, pain, obstipation, diarrhoea, dyspnoea, angina pectoris, weight loss > 2 kg *during the last month*; falls requiring medical treatment, fractures, anaemia, gastrointestinal bleeding, cardiovascular problems, hospitalisation *during the last 12 months*; these parameters were collected for describing the study sample at baseline respectively for patient monitoring throughout the study.
- Results of the questionnaires: EQ-5D-5L, 5-GDS, 6-CIT (Table 1)

At the planned study visits after 8, 16 and 24 months (*follow-up 1 = FU1*, *follow-up 2 = FU2* and *final examination = T*_*2*_), the same data were collected (except EQ-5D-5L/5-GDS/6-CIT: only at T_0_ and T_2_), and additionally the following events/parameters:

- Death
- Non-elective hospital admissions
- Only in the intervention group: the number and types of experts’ recommendations and the reactions of the GPs (adoption or non-adoption of the recommendations, explanation in case of non-adoption, number and kinds of stopped drugs, not discontinued drugs despite the recommendation of discontinuation, re-prescription of a stopped medication).

All outcome measures (Table 1) were recorded by the GPs when they occurred or at least at the planned follow-up study visits. The GPs were also free to conduct additional visits according to their discretion. The GPs were informed about the events of interest (death, hospital admission, falls, fractures) by the patients’ anamnesis during the study visits or other patient contacts within the study period, or by information of the patients’ relatives. Also discharge letters from hospitals and/or contacts with hospital physicians or other specialists served as source of information for the GPs regarding patient-related events.

All patient data were pseudonymised by the GPs (using anonymous patient numbers) and afterwards exported by the research team for statistical analysis.

### Monitoring

All CRFs were controlled by the project team. In case of incompleteness, the GPs were contacted to retrieve missing data.

As the intervention included possible discontinuation of (however not evidence-based) drugs, appearance of new symptoms was monitored thoroughly by the GPs. The GPs were informed that any drug discontinued in the study could be re-prescribed in case of symptom recurrence.

No explicit stopping rules were defined; no respective concerns to stop the study occurred throughout the study period.

### Data analysis

Statistical analysis was performed by an independent statistician at the end of the study without interim analyses using Stata 16.1. (StataCorp. 2019. College Station, TX).

Categorical variables were summarised as absolute and relative frequencies, while numerical variables as median and interquartile range (IQR).

Mann-Whitney-U tests and Fisher exact tests were used for unadjusted comparison of (continuous and categorical, respectively) baseline characteristics and secondary outcomes between the study groups.

Primary and secondary binary outcomes were also compared between groups in both uni- and multivariable settings using logistic regression (binary outcomes) and Cox regression (time-to-event outcomes) with cluster-robust standard errors of estimates which take into account intragroup cluster correlation [38] (Table 3). In the multivariable models the following baseline variables were included for adjustment: sex, age, number of conditions, number of symptoms (within 1 month before T_0_), number of falls, number of fractures and number of hospitalisations (each within 12 months before T_0_).

The composite primary endpoint was analysed according to intention-to-treat and per-protocol principles, the secondary outcomes were analysed as per-protocol (see below).

All tests were two-sided; a significance level of *p* < 0.05 was used throughout.

Baseline demographical data of GPs/patients, diagnoses and medication-related data had no missing values.

Laboratory values were not available for all patients; in case of missing values, a listwise deletion was applied, i.e. individuals with missing data were excluded from analysis of laboratory values. When T_2_ data were missing due to death or withdrawal, we used the last recorded outcome value.

Values obtained from EQ-5D-5L were converted into the EQ-5D index (single value per patient) by using the German EQ-5D-5L Crosswalk Value Set [39] as no country-specific value set was available for Italy [40] and Germany most closely approximates to the investigated northern Italian region^3^ [41].

## Results

### Study participants

Of 270 invited GPs, *n* = 43 (15.9%) participated. The 43 GPs had 71,014 enrolled patients overall and 8015 patients aged ≥75 years. Out of these, 1075 patients (13.4%) were on therapy with ≥8 drugs and thus eligible.

The 43 GPs recruited *n* = 579 patients (53.9% of the eligible patients). After cluster-randomisation, 22 GPs and 307 patients were allocated to the IG, 21 GPs and 272 patients to the CG. 94 patients (IG: 57, CG: 37) were lost to follow-up because of death or withdrawal (Fig.1).

Median age of all participating patients was 81 years (IQR 78–85), 60.3% were female. Median drug use was 9 (IQR 8–11) drugs, median number of diagnoses was 5 (IQR 4–6).

Most baseline characteristics were well-balanced between IG and CG (age, gender, number of chronic conditions, laboratory parameters, health-related quality of life, cognitive function, affective status, number of pre-interventional fractures, frequency of PIMs and DDIs). Pre-interventional hospitalisations, falls and symptoms were significantly more frequent in the IG while the median number of drugs was significantly higher in the CG (Table 2).

**Table 2:** Baseline characteristics of the participating patients (n total = 579)

Characteristics patients		Intervention group (*n* = 307)	Control group (*n* = 272)	***p***-value
**Age**				
Median (IQR)		81 (78–85)	81 (79–85)	0.413 ^i^
**Gender**				
Female: n (%)		180 (58.6%)	169 (62.1%)	0.396 ^ii^
**Drug prescriptions**				
Total number of drugs		2914	2700	–
Median number of drugs (IQR)		9 (8–10)	9 (8–11)	**0.006** ^i^
**Most frequently prescribed drug classes** **[number of patients concerned per drug class]**				
ARBs, ACE-inhibitors		256 (134, 122)	225 (117, 108)	**–**
PPIs		161	159	**–**
Statins		163	156	**–**
Platelet-aggregation inhibitors		167	144	**–**
Beta-blockers		159	147	**–**
Minor diuretics (predominantly hydrochlorothiazide)		153	120	**–**
CCBs		133	115	**–**
Loop diuretics (predominantly Furosemide)		126	118	**–**
Vitamins (predominantly vit. D)		118	109	**–**
Antidepressants, antipsychotics		117	98	**–**
Oral anticoagulants		100	90	**–**
Anxiolytics/hypnotics (Benzodiazepines, Zolpidem)		79	94	**–**
Dietary supplements (predominantly Calcium)		86	85	**–**
Oral antidiabetic drugs		85	71	**–**
Antiasthmatic agents, beta-adrenergics, anticholinergics		71	63	**–**
**Chronic conditions**				
Total number of chronic diseases		1664	1479	–
Median number of chronic diseases (IQR)		5 (4–6)	5 (4–6)	0.346 ^i^
**EQ-5D-5L index**				
Median (IQR)		0.815 (0.710–0.910)	0.810 (0.716–0.909)	0.762 ^i^
**EQ-VAS score**				
Median (IQR)		60.0 (50.0–80.0)	65.0 (50.0–78.8)	0.581 ^i^
**5-GDS:** Score ≥ 2 points (affective impairment)				
n patients (%)		94 (30.6%)	76 (27.9%)	0.522 ^ii^
**6-CIT:** Score ≥ 8 points (cognitive impairment)				
n patients (%)		77 (25.1%)	81 (29.8%)	0.225 ^ii^
**Biometric and laboratory parameters**	Unit			
BMI: Median (IQR)	kg/m^2^	26.7 (23.9–29.7)	26.2 (23.5–29.4)	0.522 ^i^
Creatinine: Median (IQR)	mg/dl	1.1 (0.9–1.3)	1.0 (0.8–1.4)	0.307 ^i^
**Number of events within last 12 months before T**_**0**_		**Intervention group** (*n* = 307)	**Control group** (n = 272)	**p-value**
Patients with ≥1 hospitalisation (% of patients)		85 (27.7%)	37 (13.6%)	< **0.001**^ii^
Patients with ≥1 falls requiring medical treatment (%)		66 (21.5%)	28 (10.3%)	< **0.001**^ii^
Patients with ≥1 fractures (%)		23 (7.5%)	11 (4.0%)	0.110 ^ii^
**Symptoms within 1 month before T**_**0**_^**§**^		**Intervention group** (n = 307)	**Control group** (n = 272)	**p-value**
Total number of symptoms		358	193	**–**
Patients with ≥1 symptom (%)		173 (54.4%)	107 (39.3%)	**< 0.001**^ii^
Median (IQR)		1 (0–2)	0 (0–1)	< **0.001** ^i^
**PIMs and DDIs**		**Intervention group** (n = 307)	**Control group** (n = 272)	**p-value**
**n drugs Beers list** [32]				
Total number (% of total prescriptions)		181 (6.2%)	160 (5.9%)	–
n patients with ≥1 Beers-listed drug		142 (46.3%)	124 (45.6%)	0.993^ii^
Median (IQR)		0 (0–1)	0 (0–1)	0.993 ^i^
**D or X drug-drug interactions** ^**§§**^ [34]				
Total number		396	380	–
n patients with ≥1 D/X drug-drug interaction (%)		203 (66.1%)	188 (69.1%)	0.477^ii^
Median (IQR)		1 (0–2)	1 (0–2)	0.424 ^i^

^i^ Mann-Whitney U test, ^ii^ Fisher exact test

^§^ The following symptoms were considered: nausea, vertigo, pain, obstipation, diarrhoea, dyspnoea, angina pectoris, weight loss ≥2 kg; full list: Supplementary Tab.I

^§§^ Drug-drug interactions: category D = consider drug modification, category X = avoid combination [34]

*IQR* Interquartile range, *EQ-5D* 5-Item questionnaire measuring health-related quality of life, *VAS* Visual analogue scale, *5-GDS* 5-Item Geriatric Depression Scale, *6-CIT* 6-Item Cognitive Impairment Test, *BMI* Body mass index, *PIMs* Potentially inappropriate drugs, *DDIs* Drug-drug interactions

### Primary endpoint and secondary endpoints

For the intention-to-treat analysis (ITT) of the primary endpoint, participants who were lost to follow-up were included as having reached the outcome (Table 3).

**Table 3:** Results of primary and secondary outcomes at T_2_ (n total = 579)

Composite primary outcome	IG	CG	Unadjusted OR (95% CI) ^**§**^	***p***-value	Adjusted OR (95% CI) ^**§**^	***p***-value
**First non-elective hospital admission or death**	**n = 307**	***n*** **= 272**				
ITT analysis (including patients lost to FU as having reached the outcome)	151 (49.2%)	102 (37.5%)	1.7 (1.13–2.54)	**0.01** ^i^	1.46 (0.99–2.18)	0.06 ^iii^
**First non-elective hospital admission or death**	**n = 281**	***n*** **= 257**				
PP analysis (excluding patients lost to FU: IG n = 26, CG n = 15)	125 (44.5%)	87 (33.9%)	1.57 (1.01–2.43)	**0.04** ^i^	1.33 (0.87–2.04)	0.2 ^iii^
**Secondary outcomes**	**IG**	**CG**	**Unadjusted HR (95% CI)** ^**§**^	**p-value**	**Adjusted HR (95% CI)** ^**§**^	**p-value**
**Mortality**	**n = 281** ^§§^	**n = 257** ^§§^				
Post-interventional death (all-cause)	31 (11.0%)	22 (8.6%)	1.54 (0.74–3.25)	0.25 ^iii^	1.34 (0.62–2.91)	0.46 ^iii^
**Secondary outcomes**	**IG**	**CG**	**Unadjusted OR (95% CI)** ^**§**^	**p-value**	**Adjusted OR (95% CI)** ^**§**^	**p-value**
**Mortality**	**n = 281** ^§§^	**n = 257** ^§§^				
Post-interventional death (all-cause)	31 (11.0%)	22 (8.6%)	1.32 (0.61–2.86)	0.5 ^i^	1.18 (0.51–2.72)	0.7 ^iii^
**Non-elective hospital admissions**	**n = 281**	**n = 257**				
Patients with ≥1 hospital admission (%)	103 (36.7%)	68 (26.5%)	1.61 (1.03–2.51)	**0.04** ^i^	1.39 (0.95–2.03)	0.09 ^iii^
Total number of hospital admissions	122	74	–	–	–	–
Median (IQR)	0 (0–1)	0 (0–1)	–	**0.008** ^ii^	–	–
**Falls**	**n = 281**	**n = 257**				
Patients with ≥1 falls (%)	58 (20.6%)	73 (28.4%)	0.66 (0.35–1.21)	0.2 ^i^	0.55 (0.31–0.98)	**0.04** ^iii^
Total number of falls	65	76	–	–	–	–
Median (IQR)	0 (0–0)	0 (0–1)	–	**0.047** ^ii^	–	–
**Fractures**	**n = 281**	**n = 257**				
Patients with ≥1 fractures (%)	25 (8.9%)	13 (5.1%)	1.83 (0.87–3.86)	0.1 ^i^	1.61 (0.8–3.2)	0.2 ^iii^
Total number of fractures	26	13	–	–	–	–
Median (IQR)	0 (0–0)	0 (0–0)	–	0.08 ^ii^	–	
**Number of drug prescriptions**	**n = 250** ^§§^	***n*** **= 235** ^§§^		***p*-value**	**–**	**p-value**
Total number of drugs	2218	2140	–	–	–	–
Median number of drugs (IQR)	8 (7–10)	9 (8–10)	–	0.1 ^ii^	–	–
Change in number of drugs from baseline: median reduction (IQR)	0 (−1–0)	0 (−2–0)	–	0.6 ^ii^	–	–
**EQ-5D-5L index**	**n = 250**	**n = 235**		***p*-value**	**–**	***p*-value**
Median (IQR)	0.806 (0.698–0.909)	0.806 (0.701–0.909)	–	0.3 ^ii^	–	–
**EQ-VAS score**	**n = 250**	***n*** **= 234**		**p-value**	**–**	**p-value**
Median (IQR)	60 (50–75)	60 (50–70)	–	0.2 ^ii^	–	–
**5-GDS:** Score ≥ 2 points (affective impairment)	**n = 250**	**n = 235**		**p-value**	**–**	**p-value**
n patients (%)	105 (42.0%)	81 (34.5%)	1.38 (0.89–2.12)	0.2 ^i^	1.41 (0.9–2.21)	0.1 ^iii^
**6-CIT:** Score ≥ 8 points (cognitive impairment)	**n = 250**	**n = 235**		**p-value**	**–**	**p-value**
n patients (%)	64 (25.6%)	61 (26.0%)	0.98 (0.57–1.7)	0.9 ^i^	0.88 (0.48–1.62)	0.7 ^iii^

^i^ Fisher exact test, ^ii^ Mann-Whitney U test, ^iii^ Wald test

^§^ Event occurred / not occurred within the observation period of 24 months; adjusted by the following baseline characteristics: sex, age, number of conditions, number of symptoms within 1 month before T_0_, number of falls, number of fractures and number of hospitalisations within 12 months before T_0_

^§§^ IG *n* = 281, CG *n* = 257 after exclusion of pre-interventional deaths and withdrawals; IG *n* = 250, CG *n* = 235 after additional exclusion of post-interventional deaths due to missing values

*IG* Intervention group, *CG* Control group, *OR* Odds ratio, *CI* Confidence intervals, *ITT* Intention-to-treat analysis, *FU* Follow-up, *PP* Per-protocol analysis, *IQR* Interquartile range, *EQ-5D* 5-Item questionnaire measuring health-related quality of life, *VAS* Visual analogue scale, *5-GDS* 5-Item Geriatric Depression Scale, *6-CIT* 6-Item Cognitive Impairment Test

The secondary outcomes were analysed as per-protocol (PP) by including patients with outcome measures up to death or T_2_, excluding those participants who were lost to follow-up due to pre-interventional death or withdrawal (IG: *n* = 26, CG: *n* = 15; Fig.1).

In addition, for the outcomes number of drugs and EQ-5D-5L/5-GDS/6-CIT scores, T_2_ data were considered missing for patients with post-interventional deaths (IG: *n* = 31, CG: *n* = 22) (Table 3).

For the outcomes number of drugs and EQ-5D-5L/5-GDS/6-CIT scores, T_2_ data were *additionally* missing for post-interventional deaths (IG: n = 31, CG: n = 22) (Table 3).

*Primary outcome:* In the IG, 125 of 307 patients (40.7%) experienced the primary outcome of at least one non-elective hospitalisation or death. Moreover, 26 patients (8.5%) were lost to follow-up due to withdrawal or pre-interventional death. In the CG, 87 of 272 patients (32.0%) experienced non-elective hospital admissions or death and 15 patients (5.5%) were lost to follow-up.

In both the ITT and PP analysis, the adjusted rates of occurrence of the primary outcome in the CG and IG groups did not differ significantly (ITT: adjusted OR 1.46, 95%CI 0.99–2.18, *p* = 0.06; PP: adjusted OR 1.33, 95%CI 0.87–2.04, *p* = 0.2).

In the unadjusted analysis, the difference between the rates was statistically significant (ITT: unadjusted OR 1.7, 95%CI 1.13–2.54, *p* = 0.01; PP: unadjusted OR 1.57, 95%CI 1.01–2.43, *p* = 0.04) (Table 3).

*Secondary outcomes*: Deaths and hospitalisations as single endpoints occurred tendentially more often in the IG, hospitalisations were significantly more frequent in the IG according to the unadjusted analysis (unadjusted OR 1.61, 95%CI 1.03–2.51, *p* = 0.04) but not in the adjusted analysis (adjusted OR 1.39, 95%CI 0.95–2.03, *p* = 0.09). Regarding falls, a statistically significant risk reduction favouring the IG was observed in the adjusted analysis (adjusted OR 0.55, 95%CI 0.31–0.98; p = 0.04).

No statistically significant difference between the treatment groups was found regarding fractures, number of drugs, EQ-5D/EQ-VAS, 5-GDS and 6-CIT scores (Table 3).

### Results of the intervention

For 15.8% of all drug prescriptions, at least two experts agreed by rating them as inappropriate; thus, for these prescriptions, recommendations were elaborated and sent to the respective GP. The GPs received recommendations of drug discontinuation for 76.5% of the included patients. The EbM-expert rated 16.6% of all prescriptions as inappropriate, the clinical pharmacologist 14.1%, and the internist 13.6%. Pharmacologist and EbM-expert showed the highest agreement upon recommendations (79.7%), pharmacologist and internist the lowest (66.6%). The same three drug classes (anxiolytics/hypnotics, PPIs and beta-blockers) were valued most frequently as inappropriate by all three experts (Table 4).

**Table 4:** Experts’ valuation of patients’ drug regimens

Recommendations	Intervention group: n = 281 patients ^§^		
	n drugs	% of total prescriptions (*n* = 2658)	
Total number of drugs valued as inappropriate	419	15.8%	
		Median (IQR)	min – max
Number of inappropriate drugs per patient (n = 281)		1 (1–2)	0–6
Number of inappropriate drugs per GP (*n* = 22)		20 (15–22)	5–37
**Frequency of inappropriate drugs per patient**	n patients	% of participating patients (*n* = 281)	
n patients with 0 inappropriate drugs	66	23.5%	
n patients with 1 inappropriate drug	91	32.4%	
n patients with 2 inappropriate drugs	70	24.9%	
n patients with 3 inappropriate drugs	37	13.2%	
n patients with ≥4 inappropriate drugs	17	6.0%	
*TOTAL patients with ≥ 1 inappropriate drug*	*215*	*76.5%*	
**Frequency of inappropriate drugs per GP**	n GPs	% of participating GPs (n = 22)	
n GPs with 5–10 inappropriate drugs	3	13.6%	
n GPs with 11–20 inappropriate drugs	10	45.5%	
n GPs with > 20 inappropriate drugs	9	40.9%	
**Drug classes rated as inappropriate**	n drugs	% of prescribed drugs	% of inappropriate drugs (*n* = 419)
Anxiolytics/hypnotics (Benzodiazepines, Zolpidem)	73	92.4% (*n* = 79)	17.4%
Alpha-blockers	15	65.2% (n = 23)	3.6%
Antiarrhythmics	10	58.8% (*n* = 17)	2.4%
NSAIDs, COX-2-inhibitors (Coxibe)	21	56.8% (*n* = 37)	5.0%
PPIs	72	44.7% (*n* = 161)	17.2%
Antidepressants, antipsychotics	34	29.1% (*n* = 117)	8.1%
Oral antidiabetic drugs	22	25.9% (*n* = 85)	5.3%
Drugs for gout treatment (Allopurinol)	9	21.4% (*n* = 42)	2.2%
Corticosteroids	11	19.3% (n = 57)	2.6%
Beta-blockers	29	18.2% (*n* = 159)	6.9%
Opioids	10	14.7% (*n* = 68)	2.4%
Analgesics - Paracetamol	9	13.8% (*n* = 65)	2.2%
Dietary supplements (predominantly Calcium)	9	10.5% (*n* = 86)	2.2%
Vitamins (predominantly Vit. D)	11	9.3% (*n* = 118)	2.6%
Oral anticoagulants	9	9.0% (*n* = 100)	2.2%
**Experts’ motivations for recommendation to stop**	n drugs	% of inappropriate drugs (n = 419)	
Lack of clear indication	255	60.9%	
Contraindicated in older-aged persons	63	15.0%	
Not first choice	57	13.6%	
Not indicated as long-term therapy	27	6.4%	
Disease-specific contraindication	9	2.1%	
High risk of ADEs	3	0.7%	
High risk of significant DDIs	2	0.5%	
Unfavourable risk-benefit assessment	2	0.5%	
Duplication of drugs	1	0.2%	
**n drugs valued as inappropriate per expert**	n drugs (% of total prescriptions)	Median (IQR)	min – max
Expert of Evidence-based Medicine (EbM)	441 (16.6%)	1 (1–2)	0–6
Clinical pharmacologist	374 (14.1%)	1 (0–2)	0–6
Specialist of internal medicine	361 (13.6%)	1 (0–2)	0–6
**Most frequent inappropriate drug classes per expert**	Clinical pharmacologist	Internist	EbM-expert
Anxiolytics/hypnotics (Benzodiazepines, Zolpidem): n	72	72	73
PPIs: n	71	63	73
Beta-blockers: n	21	20	38
**Concordance between experts**	Pharm – Int	Pharm – EbM	EbM – Int
Patients where two experts fully agreed: n (%)	187 (66.6%)	224 (79.7%)	207 (73.7%)

^§^ After exclusion of pre-interventional deaths and withdrawals, the intervention was conducted on 281 patients (Fig.1)

*IQR* Interquartile range, *Min* Minimum, *Max* Maximum, *GP(s)* General practitioner(s), *PPIs* Proton pump inhibitors, *NSAIDs* Non-steroidal anti-inflammatory drugs, *COX* Cyclooxygenase, *ADEs* Adverse drug events, *DDIs* Drug-drug interactions, *Pharm* Pharmacologist, *Int* Internist, *EbM* Evidence-based Medicine

The absence of a clear indication was by far the most frequent rationale for the recommendation to stop a drug (58.7%), followed by contraindication in older age (15%), contraindication as first-line treatment (13.6%) or as long-term therapy (6.4%), and presence of condition-specific contraindications (2.1%).

The drug classes most frequently rated as inappropriate in relation to their prescribing frequency were anxiolytics/hypnotics (92.4%), alpha-blockers (65.2%), antiarrhythmics (58.8%), NSAIDs/COX-2-inhibitors (56.8%), PPIs (44.7%) and antidepressants/antipsychotics (29.1%) (Table 4).

Allopurinol (55.6%), NSAIDs/COX-2-inhibitors (42.9%), beta-blockers (34.5%), alpha-blockers (33.3%) and antiarrhythmics (30.0%) were the most frequently discontinued drug classes. Anxiolytics/hypnotics were stopped less frequently (21.9%). PPIs were withdrawn in 18.1%, antidepressants/antipsychotics in 11.8%.

The GPs discontinued 24.3% of the recommended drugs in 37.2% of the concerned patients (Table 5).

**Table 5:** Reactions of the GPs on the recommendations of the experts

Drugs rated as inappropriate and DISCONTINUED			
	n drugs (%)	Median (IQR)	min – max
Total number of stopped drugs	102 (24.3%)		
n stopped drugs per patient		0 (0–1)	0–5
n stopped drugs per GP		4.5 (1.3–7)	1–12
n patients with ≥1 stopped drug	80 (37.2%)		
**Most frequently stopped drug classes**	n drugs	% of drugs rated as inappropriate per drug class	
Drugs for gout treatment (Allopurinol)	5	55.6% (*n* = 9)	
NSAIDs, COX-2-inhibitors (Coxibe)	9	42.9% (*n* = 21)	
Beta-blockers	10	34.5% (*n* = 29)	
Alpha-blockers	5	33.3% (n = 15)	
Antiarrhythmics	3	30.0% (n = 10)	
Corticosteroids	3	27.3% (n = 11)	
Antiepileptics	4	26.7% (n = 15)	
Anxiolytics/hypnotics (Benzodiazepines, Zolpidem)	16	21.9% (*n* = 73)	
Opioids	2	20.0% (n = 10)	
Oral antidiabetic drugs	4	18.2% (n = 22)	
PPIs	13	18.1% (*n* = 72)	
Antidepressants, antipsychotics	4	11.8% (*n* = 34)	
**Drugs rated as inappropriate and NOT DISCONTINUED**			
	n drugs	% of drugs / patients	
Total number of not stopped drugs	317	75.7% (n = 419)	
n patients with ≥1 not stopped drugs	135	62.8% (*n* = 215)	
n GPs who gave an explanation for not stopping	177	55.8% (*n* = 317)	
**GPs’ explanations for not stopping an inappropriate drug**	n drugs	% of not discontinued drugs (n = 317)	
An indication for the drug was given	131	41.3%	
Prescribed/recommended by specialists, therefore not withdrawn	24	7.6%	
Patient’s refusal of discontinuation	15	4.7%	
Discontinuation attempted and failed (symptom recurrence)	5	1.6%	
Dose reduction instead of complete withdrawal	2	0.6%	
**Most frequently not stopped drug classes**	n drugs	% of drugs rated as inappropriate per drug class	
Oral anticoagulants	9	100% (n = 9)	
Vitamins (predominantly Vit. D)	10	90.9% (n = 11)	
Analgesics - Paracetamol	8	88.9% (*n* = 9)	
Dietary supplements (predominantly Calcium)	8	88.9% (n = 9)	
Antidepressants, antipsychotics	30	88.2% (n = 34)	
PPIs	59	81.9% (n = 72)	
Oral antidiabetic drugs	18	81.8% (n = 22)	
Opioids	8	80.0% (n = 10)	
Anxiolytics/hypnotics (Benzodiazepines, Zolpidem)	57	78.1% (n = 73)	
	n drugs	%	
Total number of re-prescribed drugs	35	34.3% (*n* = 102 discontinued drugs)	
n patients with ≥1 re-prescribed drug	33	41.2% (*n* = 80 patients with ≥1 stopped drug)	
n patients with 0 re-prescribed drugs	47	58.8% (n = 80 patients with ≥1 stopped drug)	
**Most frequently re-prescribed drug classes**	n drugs	% of stopped drugs per drug class	
Benzodiazepines	7	43.8% (n = 16)	
PPIs	6	46.2% (*n* = 13)	
NSAIDs, COX-2-inhibitors (Coxibe)	4	44.4% (n = 9)	
Beta-blockers	4	40.0% (n = 10)	
Antidepressants	2	50.0% (n = 4)	

*IQR* Interquartile range, *Min* Minimum, *Max* Maximum, *GP(s)* General practitioner(s), *NSAIDs* Non-steroidal anti-inflammatory drugs, *COX* Cyclooxygenase, *PPIs* Proton pump inhibitors

The most frequently not discontinued drugs/drug classes were oral anticoagulants (100%), Vitamin D (90.9%), Paracetamol and Calcium (88.9% each); however, these drugs had been indicated as inappropriate only in single cases. Among the most frequently not stopped medications, also drug classes with more frequent inappropriate ratings were represented: antidepressants/antipsychotics (88.2%), PPIs (81.9%) and anxiolytics/hypnotics (78.1%) (Table 5).

For 55.8% of the 317 not discontinued inappropriate drugs, the GPs provided an explanation why they did not withdraw the drug. The most frequent reason was a given indication (41.3%); prescription by specialists (7.6%), patient’s refusal (4.7%) and symptom recurrence (1.6%) were less frequently mentioned (Table 5).

35 (34.3%) of the 102 discontinued drugs were re-introduced: 40–50% of the withdrawn benzodiazepines, PPIs, NSAIDs, beta-blockers and antidepressants. Thus, *definitive* discontinuation was obtained for 67 drugs (16.0% of drugs rated as inappropriate) respectively in 47 patients (58.8% of the patients with discontinued drugs during the study) (Table 5)

## Discussion

### Endpoints

This northern-Italian RCT investigated the effect of medication reviews and recommendations provided by three experts with different professional background on patient-relevant outcomes in older-aged general practice patients on polypharmacy. The study found a high prevalence of polypharmacy, PIMs and DDIs, as described in the publication of epidemiological baseline data [31]. The composite primary outcome of non-elective hospital admissions or death was experienced significantly more often in the IG in the unadjusted analysis; yet, as significance disappeared after adjustment, which was also noted for hospitalisations as single secondary endpoint, this phenomenon seems to be strongly related to the higher occurrence of hospital admissions in the IG within the pre-interventional period. Also, the frequency of pre-interventional falls and symptoms was significantly higher in the IG. Thus, patients of the IG seemed to be in less favourable physical preconditions than CG patients. This phenomenon could have been entailed by the cluster randomisation (e.g., GPs of the IG could have systematically recruited more clinically impaired patients); however, the cluster effects were considered in the outcome analysis and did not significantly influence the results.

Hospitalisation rates (as secondary endpoint) remained higher in the IG than in the CG at T_2_, yet, the difference between the study groups was reduced compared to baseline. For both groups, the descriptive within-group analysis showed an increase of hospitalisations up to T_2_, whereby the increase in the CG was more pronounced and nearly doubled (Supplementary Tab.III). Therefore, although the intervention was not able to actually reduce mortality and hospitalisation rates, it may cautiously be interpreted as having demonstrated a positive impact in terms of a slowed increase of hospitalisations in a frail older-aged population with natural tendency to deterioration of clinical and physiological functions.

No significant difference was detected regarding mortality as single endpoint and fractures. Both outcomes occurred tendentially but not significantly more often in the IG, probably because of the higher clinical impairment of IG patients at baseline.

The assessed patient-reported outcomes did not significantly differ between IG and CG either. In both treatment groups, quality of life and affective status showed a tendency to decrease over time, most probably due to the natural functional decline in older-aged patients. Interestingly, the cognitive function of the assessed participants remained stable throughout the observation period which concords to the fact that only few patients with diagnosticated dementia participated in the study [31] and severe cognitive impairment was an exclusion criterium.

A positive result was by contrast found regarding falls: although being more than twice as frequent in the IG at baseline, a significant risk reduction favouring the IG was observed at the end of the study. Therefore, the intervention seemed to have significantly reduced falls in the investigated older-aged population. On the other hand, as a rather small part of the medications rated as inappropriate was actually withdrawn (see below), the extent of the real impact of the intervention on the measured outcomes should not be overestimated. Yet, our results may suggest that even modest reductions of inappropriate medications are able to entail clinical benefits, and to do so without negative impact on measured patient-related outcomes. This was confirmed also by the European multicenter trial PRIMA-eDS [42] and points to safety of deprescribing [27, 43]. True improvements of patient-related outcomes, especially mortality and hospital admissions, may be difficult to be achieved in older-aged multimorbid populations with natural tendency to functional deterioration [22]; therefore, besides from real measurable improvements, also stabilisation of clinical outcomes could be considered a positive result. Moreover, the medical impact of reduced falls should not be underestimated.

Although it was significantly higher in the CG at baseline, the median number of drugs did not differ between the study groups at T_2_. In both groups, the number of drugs decreased over time while the median reduction was tendentially higher in the CG. Thus, in contrast to the findings of the PRIMA-eDS trial [42], the intervention in our study did not have a clear impact on the number of prescriptions. This is not surprising as discontinuation of inappropriate drugs was carried out only in 37% of concerned patients and only 16% of the drugs recommended to discontinue were definitively withdrawn by the GPs; a notable reduction of the overall number of drugs could therefore not be expected. On the other hand, physicians of the CG could have changed their prescribing behaviour as well due to the awareness of participating in a study aiming at reducing inappropriate polypharmacy (study effect); this might have contributed to the even higher reduction of drug prescriptions in the CG.

In the available literature, a persisting paucity is noted of studies investigating the reduction of polypharmacy in daily practice [11]; this applies especially to high-grade evidence and proven effects on patient-relevant outcomes. A British RCT with a comparable intervention in care homes largely confirmed our results: the study found a significant reduction in number of falls, but no change in number of drugs, hospitalisations, mortality, cognitive function and activities of daily living [44]. The PRIMA-eDS study found no conclusive evidence for the reduction of mortality, non-elective hospitalisations, falls, fractures or improvements in quality of life (SF-12 physical and mental component scores) while the number of drugs was significantly reduced without negative impact on patient outcomes [42].

Also other studies in the primary care setting aiming at decreasing inappropriate polypharmacy achieved significant reductions of drug numbers [45–48]. However, a recent update of a Cochrane review [23] found no clear evidence that the assessed interventions were able to reduce the number of inappropriate prescriptions, hospital admissions, medication-related problems, or to enhance quality of life [49]. These results confirmed those derived from a former systematic review and meta-analysis [50]. Positive impacts of deprescribing interventions on all-cause mortality were found for non-randomised studies [4], but convincing evidence from randomised trials is lacking [51].

### Intervention

15.8% of all drug prescriptions in our sample were valued as inappropriate by at least two experts (median: one per patient). This number appears rather modest, however, more than three quarters of the patients were treated with at least one inappropriate drug and nearly one fifth received three or more inappropriate drugs. The EbM-expert valued the highest proportion of drugs as inappropriate (16.6%), while the internist who was the expert most closely related to real practice rated the lowest proportion of drugs as inappropriate (13.6%).

In relation to their prescribing frequency, the most concerned drug classes in our cohort were anxiolytics/hypnotics, alpha-blockers, antiarrhythmics, NSAIDs/COX-2-inhibitors, PPIs and antidepressants/antipsychotics. Among these, antidepressants/antipsychotics, PPIs and anxiolytics were the most difficult to discontinue whereas the largest potential of deprescribing was observed for Allopurinol and NSAIDs. As previous literature shows, NSAIDs belong to those drug classes causing the majority of drug-related hospital admissions [52]. Thus, a careful consideration of their risk and benefit may contribute to avoiding preventable hospitalisations. Yet, although NSAIDs were among the most successfully discontinued inappropriate medications in our sample, they were withdrawn in only 43% of those cases where discontinuation was recommended.

In total, 24.3% of the recommended drugs were stopped by the GPs. Of these, a third was restarted due to re-occurrence of conditions or symptoms; this concerned mainly antidepressants, PPIs, NSAIDs, benzodiazepines and beta-blockers. Thus, in total, effective withdrawal was obtained only for 16% of the recommended drugs. A narrative review found lower general proportions of patients who needed to restart discontinued drugs (2–18%) while the success rates of definitive discontinuation differed largely across drug classes (14–64% for PPIs, 25–85% for benzodiazepines) [28]. A non-controlled pre-post study involving community-dwelling older adults found that 82% of inappropriate drugs were withdrawn (benzodiazepines even almost 100%, PPIs 75%) and only 2% of the stopped drugs had to be re-administered. These numbers indicate a largely higher discontinuation rate than in our study, however, the study sample was small [53].

In general, the fact that many of the recommendations were not adopted by the GPs and only less than a fifth of the inappropriate medications was definitively discontinued make a conclusive statement regarding the effect of the intervention difficult. Other studies achieved higher acceptance of experts’ recommendations by the GPs (44–58%) [44, 48], however, also these numbers indicate that the implementation of such interventions meets significant barriers. This is a relevant result itself which poses the question why it is so difficult to discontinue drug therapies in patients with polypharmacy and which factors impede efficient deprescribing.

In our cohort, the most prevalent reason for recommending discontinuation by the experts was missing indication; on the other hand, most of those GPs who gave a justification for non-adherence to the experts’ recommendations reported that a specific indication was given. These contradicting points of view may have intrinsically lowered the potential for deprescribing; however, the high baseline prevalence of PIMs (46.3%) and major DDIs (66.1%) underpin the a priori-necessity of deprescribing. Besides from true missing indications or symptoms/conditions falsely interpreted by GPs as correct indications, also other scenarios could have played a role: e.g. if a GP was aware of a condition and had treated it correctly but not listed the respective diagnosis in the EHR [54]. This would represent rather a problem of thorough documentation which is however an important precondition for high-quality therapy, especially in case of changing physicians or care providers. In this way, although this was not an explicit trial objective, the study could have contributed to enhance physicians’ awareness towards consistent documentation.

Other frequent reasons for not discontinuing medications were prescriptions by specialists and patient’s refusal; these were identified also by previous studies as major barriers to deprescribing [28].

In general, the literature distinguishes three types of factors which hinder deprescribing. *Physician-related barriers* comprise lack of knowledge [55], low awareness regarding identification of inappropriate drugs, inertia (failure to act despite of awareness), or low perceived self-efficacy (e.g. GPs not ‘daring’ to stop a medication initiated by a specialist) [28]. *System-related barriers* are lack of resources and time, multiple care providers with poor collaboration among different care levels, lack of guidelines for older multimorbid patients, and missing financial incentives for GPs addressing polypharmacy [28, 56]. Although studies revealed willingness to deprescribing among patients [57, 58], also *patient-related barriers* were identified: convictions regarding necessity of drugs [28, 59], satisfaction with the current therapy [57], fears of health deterioration [28, 57, 58], free prescriptions, older age, and patients’ lower educational level [56].

A remarkable *impact towards deprescribing* was attributed to the GPs’ recommendation to stop a drug, the possibility of discussing doubts with the GP [28], a good patient-physician-relationship, and the feeling that deprescribing would be safe [57]. Moreover, multidisciplinary approaches [56], guidelines for deprescribing (e.g. a deprescribing algorithm for PPIs is available) [60], and appropriate information of patients regarding risk and benefit of stopping drugs [56] were mentioned as facilitators to deprescribing.

For improving the success of deprescribing initiatives in daily practice, these findings indicate the need of enhancing physicians’ awareness towards inappropriateness of drugs and deprescribing, of providing appropriate time and financial resources for enabling the physicians to conduct effective and satisfying deprescribing conversations with their polypharmacy patients, of strengthening the GP-patient-relationship and the physicians’ skills regarding shared decision-making, and of well-designed patient guidelines to enhance patient knowledge [58, 61]. In our study, although GPs received tailored supervision throughout the study period, patients were not directly approached e.g. by educational initiatives. Moreover, also GPs were not explicitly trained towards polypharmacy and deprescribing. Perhaps a more active inclusion of patients in the intervention and an additional pre-interventional training of the GPs could have entailed a more effective implementation.

Our results also support previous conclusions [7, 62] that prevention of polypharmacy may be more successful than an afterwards deprescribing of drugs which patients (and physicians) are used to. Thus, future interventions should additionally focus on new prescriptions. In daily practice, besides from medication reviews performed by physicians, pharmacists or multidisciplinary teams, also electronic tools providing decision support in real time may be useful (and even more practicable) for this purpose [42].

### Strengths and limitations

A strength of the study is that we enrolled patients aged ≥75 years, as the older age groups are less studied up to now although being the most vulnerable cohort of patients [5].

A further strength is the multidisciplinary approach, i.e. the involvement of experts from three different fields of specialisation with the need of concordance of at least two experts. The experts’ recommendations were intended as an aid to the shared decision-making process between physicians and patients, not to replace clinical judgement and individual patient counselling.

Moreover, the close involvement of GPs and patients in the intervention can be considered a strength [28] as well as the integration of the intervention in daily practice; however, at the same time, this made its implementation challenging and probably met several barriers which we were not able to fully identify neither to confront. Deprescribing addressed only withdrawal of drugs; a more sensitive approach could be achieved by also recommending dose reduction, safer alternative drugs or starting appropriate drugs. However, the aim of this study was merely to assess the effect of drug discontinuation.

The calculated sample size was low as clustering had not been considered. This led to consequences for the investigation of the study hypotheses, as only falls were significantly reduced in the intervention group, and it cannot be fully excluded that the intervention could not have entailed a significant impact on the primary endpoint or on other secondary outcomes when using a cluster-considering sample size. However, despite of the failed achievement of statistical significance for most of the endpoints, conclusions can be drawn from the *significant* results (reduction of falls). Moreover, as mentioned above, several previous studies using comparable interventions found similar results in terms of missing impact on mortality and hospitalisations; thus, the inadequate sample size might probably have been not the only or not the primary cause for not achieving significance in the primary endpoint.

The documentation of the time of occurrence was complete only for mortality while for the other patient-related events (hospitalisations, falls, fractures) considerable documentation gaps emerged. Therefore, a use of time-to-event analyses was not feasible for these outcomes nor for the primary endpoint. By using binary outcomes the *rates* of occurrence are investigated, but not potential differences regarding the *time* of occurrence; this leads to an information loss which has to be considered another limitation of the study.

OTC-medications were not included in the analysis because the electronic data extraction was possible only for *prescribed* drugs which were the only drugs recorded in the EHRs. OTC-drugs only could have been collected by questioning the participating patients; however, older-aged patients not always remember all drugs they are taking and brown bag medication reviews with each patient were not feasible within the logistic constraints of the study and have also shown limitations of accuracy [63]. Thus, a reliable and complete determination of OTC-drugs was not possible in this study and was therefore a priori excluded.

However, as in Italy most continuously taken drugs are only available on prescription, the exclusion of OTC-drugs should not have entailed a substantial bias. We also excluded PRN-medications, thus, some drugs possibly interacting with diseases or other medications could have been missed as well.

We did not evaluate the specific causes for hospitalisations and mortality e.g. differentiation between ADE-related events or other causes. Quantifying the number of *drug-related* events could have provided a better description of the potential link between polypharmacy exposure and hospitalisations respectively mortality.

Inter-rater agreement between experts’ recommendations was relatively high (≥66%) and the same three drug classes were most frequently valued as inappropriate by all experts. However, these findings also indicate that experts’ appraisal and recommendations regarding drugs vary to some extent depending on the professional and clinical background.

We did not assess the GPs’ experience or satisfaction regarding the intervention. Yet, this could be an interesting subject for future studies to e.g. qualitatively investigate the GPs’ experiences about a similar intervention and thus to possibly improve its degree of implementation.

Blinding of study participants was not possible due to the nature of the intervention. Yet, allocation concealment was assured as baseline data were collected before randomisation.

Inhomogeneity of some baseline characteristics across the study groups was not avoidable due to the size of the study sample and because of heterogeneity among patients and practices. Randomisation at a patient level could probably help to achieve a better balance of baseline covariates between the study groups, nevertheless, cluster-randomisation in our study was necessary to avoid contamination effects.

Both the GP and the patient sample were consecutively recruited to reduce the risk of selection bias. Yet, we enrolled only community-living patients who visited the GP office. The GP sample was small and thus not fully representative. Generalisability is also limited by the fact that our findings derive from a specific Italian region and patterns of polypharmacy might differ in other countries, as well as in populations with different baseline characteristics (e.g. with high-grade cognitive impairment). However, as stated above, our results are confirmed by other studies with comparable interventions deriving from different European countries; thus, we postulate that our results and implications might be applicable also to other national circumstances.

## Conclusion

Definitive discontinuation was feasible for only one out of six inappropriate medications and for one out of three patients with inappropriate drugs. Nevertheless, a significant reduction in the number of falls was noted while other outcomes (mortality and acute hospitalisation in combination and as single endpoints, number of drugs, number of fractures, quality of life, affective status and cognitive function) were not significantly altered. Thus, our results highlight the importance of optimisation of drug therapies in older-aged patients and show that also a limited reduction of inappropriate medications can lead to positive effects without a distinct increase of undesired events.

An important finding is the low implementation rate of deprescribing suggestions and the relatively high rate of restarted drugs.

This may indicate that training for GPs about controlled and effective deprescribing could be beneficial.

Our findings point out that real improvements of patient-related end-result outcomes like mortality and hospital admissions may be hardly achievable in older-aged populations with multiple conditions. Thus, for future interventions aiming at reducing inappropriate polypharmacy, we recommend that it should be questioned if stabilisation of clinical parameters would be a more appropriate outcome goal than real improvements; this could be realised e.g. by using a non-inferiority study design.

## Supplementary Information

**Additional file 1:.** The additional file contains the Supplementary Tables I-IV (word format), which report additional results information: Biometric and laboratory values at T_0_, list and frequency of symptoms at T_0_ and T_2_. Most common Beers-listed drug classes, drug-drug interactions (DDIs), and drug classes involved in DDIs at T_0_. Descriptive within group-analysis (longitudinal analysis T_0_ – T_2_) for the intervention group and the control group. Depiction of the case report form used for collection of data.

## Data Availability

The datasets used during the current study are available from the corresponding author on reasonable request.

## Abbreviations

*ACE*: Angiotensin converting enzyme
*ADE*: Adverse drug event
*ARBs*: Angiotensin II receptor antagonists
*BMI*: Body mass index
*BNP/NT-proBNP*: brain natriuretic peptide
*CCBs*: Calcium channel blockers
*CG*: Control group
*CI*: Confidence interval
*6-CIT*: 6-Item Cognitive Impairment Test
*COX*: Cyclooxygenase
CRF: Case report form
*DDIs*: drug-drug interactions
*EbM*: Evidence-based Medicine
*eDS*: Electronic decision support
*EHR(s)*: Electronic health records
*EQ-5D-5L / EQ-VAS*: 5-level EQ-5D version (5-Item questionnaire and visual analogue scale measuring health-related quality of life and self-reported health status)
*FU*: Follow-up
*5-GDS*: 5-Item Geriatric Depression Scale
*GP*: General practitioner
*ICD-10*: International Classification of Diseases, Tenth Revision
*IG*: Intervention group
*INR*: International Normalised Ratio
*IQR*: Inter-Quartile-Range
*ITT*: Intention-to-treat analysis
*NSAIDs*: Non-steroidal anti-inflammatory drugs
*OR*: Odds ratio
*OTC-drugs*: Over the counter medications (available without prescription)
*PIM*: Potentially inappropriate medication
*PP*: Per-protocol analysis
*PPIs*: Proton pump inhibitors
*PRIMA*: Polypharmacy in chronic diseases – Reduction of Inappropriate Medication and Adverse drug events in older populations (study acronym)
*PRN-drugs*: Pro re nata (as needed) medication
*QoL*: Quality of life
*RCT*: Randomised controlled trial

## Footnotes

1 In contrast to the European multicenter trial PRIMA-eDS [30], which used the same inclusion criteria and outcomes as in our study and investigated the impact of an electronic decision support system providing a comprehensive medication review, the here presented PRIMA study was limited to a northern Italian region and applied expert-based medication reviews as intervention. The data generated by the independent Italian PRIMA study were *not* part of the PRIMA-eDS trial.

2 The GPs were instructed not to order laboratory examinations specifically for the trial (except creatinine) but to use the latest record available in the electronic health record.

3 The EQ-5D user guide recommends using value sets of a country that most closely approximates to the countries of interest in case of absence of a country-specific value set [41].

## References

[1] Turnheim K (2004). Drug therapy in the elderly. Exp Gerontol.

[2] Muth C, Blom JW, Smith SM, Johnell K, Gonzalez-Gonzalez AI, Nguyen TS (2019). Evidence supporting the best clinical management of patients with multimorbidity and polypharmacy: a systematic guideline review and expert consensus. J Intern Med.

[3] Mangin D, Sweeney K, Heath I (2007). Preventive health care in elderly people needs rethinking. BMJ..

[4] Garfinkel D, Zur-Gil S, Ben-Israel J (2007). The war against polypharmacy: a new cost-effective geriatric-palliative approach for improving drug therapy in disabled elderly people. Isr Med Assoc J.

[5] Franchi C, Tettamanti M, Pasina L, Djignefa CD, Fortino I, Bortolotti A (2014). Changes in drug prescribing to Italian community-dwelling elderly people: the EPIFARM-elderly project 2000-2010. Eur J Clin Pharmacol.

[6] Tinetti ME, Bogardus ST, Agostini JV (2004). Potential pitfalls of disease-specific guidelines for patients with multiple conditions. N Engl J Med.

[7] Schuler J, Duckelmann C, Beindl W, Prinz E, Michalski T, Pichler M (2008). Polypharmacy and inappropriate prescribing in elderly internal-medicine patients in Austria. Wien Klin Wochenschr.

[8] Slabaugh SL, Maio V, Templin M, Abouzaid S (2010). Prevalence and risk of polypharmacy among the elderly in an outpatient setting: a retrospective cohort study in the Emilia-Romagna region. Italy Drugs Aging.

[9] Maher RL, Hanlon J, Hajjar ER (2014). Clinical consequences of polypharmacy in elderly. Expert Opin Drug Saf.

[10] Hanlon JT, Artz MB, Pieper CF, Lindblad CI, Sloane RJ, Ruby CM (2004). Inappropriate medication use among frail elderly inpatients. Ann Pharmacother.

[11] Koper D, Kamenski G, Flamm M, Bohmdorfer B, Sonnichsen A (2013). Frequency of medication errors in primary care patients with polypharmacy. Fam Pract.

[12] Onder G, Pedone C, Landi F, Cesari M, Della Vedova C, Bernabei R (2002). Adverse drug reactions as cause of hospital admissions: results from the Italian Group of Pharmacoepidemiology in the elderly (GIFA). J Am Geriatr Soc.

[13] Pasina L, Brucato AL, Falcone C, Cucchi E, Bresciani A, Sottocorno M (2014). Medication non-adherence among elderly patients newly discharged and receiving polypharmacy. Drugs Aging.

[14] Onder G, Bonassi S, Abbatecola AM, Folino-Gallo P, Lapi F, Marchionni N (2014). High prevalence of poor quality drug prescribing in older individuals: a nationwide report from the Italian Medicines Agency (AIFA). J Gerontol A Biol Sci Med Sci.

[15] Frazier SC (2005). Health outcomes and polypharmacy in elderly individuals: an integrated literature review. J Gerontol Nurs.

[16] Flaherty JH, Perry HM, Lynchard GS, Morley JE (2000). Polypharmacy and hospitalization among older home care patients. J Gerontol A Biol Sci Med Sci.

[17] Pirmohamed M, James S, Meakin S, Green C, Scott AK, Walley TJ (2004). Adverse drug reactions as cause of admission to hospital: prospective analysis of 18 820 patients. BMJ..

[18] Fabbietti P, Di Stefano G, Moresi R, Cassetta L, Di Rosa M, Fimognari F (2018). Impact of potentially inappropriate medications and polypharmacy on 3-month readmission among older patients discharged from acute care hospital: a prospective study. Aging Clin Exp Res.

[19] Leelakanok N, Holcombe AL, Lund BC, Gu X, Schweizer ML. Association between polypharmacy and death: A systematic review and meta-analysis. J Am Pharm Assoc (2003). 2017;57(6):729–38 e10. 10.1016/j.japh.2017.06.002 28784299

[20] Midao L, Giardini A, Menditto E, Kardas P, Costa E (2018). Polypharmacy prevalence among older adults based on the survey of health, ageing and retirement in Europe. Arch Gerontol Geriatr.

[21] Junius-Walker U, Theile G, Hummers-Pradier E (2007). Prevalence and predictors of polypharmacy among older primary care patients in Germany. Fam Pract.

[22] Mahlknecht A, Krisch L, Nestler N, Bauer U, Letz N, Zenz D (2019). Impact of training and structured medication review on medication appropriateness and patient-related outcomes in nursing homes: results from the interventional study InTherAKT. BMC Geriatr.

[23] Patterson SM, Cadogan CA, Kerse N, Cardwell CR, Bradley MC, Ryan C (2014). Interventions to improve the appropriate use of polypharmacy for older people. Cochrane Database Syst Rev.

[24] Crotty M, Halbert J, Rowett D, Giles L, Birks R, Williams H (2004). An outreach geriatric medication advisory service in residential aged care: a randomised controlled trial of case conferencing. Age Ageing.

[25] Kaur S, Mitchell G, Vitetta L, Roberts MS (2009). Interventions that can reduce inappropriate prescribing in the elderly: a systematic review. Drugs Aging.

[26] Pitkala KH, Juola AL, Kautiainen H, Soini H, Finne-Soveri UH, Bell JS (2014). Education to reduce potentially harmful medication use among residents of assisted living facilities: a randomized controlled trial. J Am Med Dir Assoc.

[27] Scott IA, Hilmer SN, Reeve E, Potter K, Le Couteur D, Rigby D (2015). Reducing inappropriate polypharmacy: the process of deprescribing. JAMA Intern Med.

[28] Reeve E, Thompson W, Farrell B (2017). Deprescribing: a narrative review of the evidence and practical recommendations for recognizing opportunities and taking action. Eur J Intern Med.

[29] Deutsche Gesellschaft für Allgemeinmedizin und Familienmedizin e.v. (DEGAM) (2012). Allgemeinmedizin - spezialisiert auf den ganzen Menschen. Positionen zur Zukunft der Allgemeinmedizin und der hausärztlichen Praxis. http://www.degam.de/files/Inhalte/Degam-Inhalte/Ueber_uns/Positionspapiere/DEGAM_Zukunftspositionen.pdf. .

[30] Sonnichsen A, Trampisch US, Rieckert A, Piccoliori G, Vogele A, Flamm M, et al. Polypharmacy in chronic diseases-Reduction of Inappropriate Medication and Adverse drug events in older populations by electronic Decision Support (PRIMA-eDS): study protocol for a randomized controlled trial. Trials. 2016;17:57. 10.1186/s13063-016-1177-8 PMC5526277 26822311

[31] Piccoliori G, Mahlknecht A, Sandri M, Valentini M, Vogele A, Schmid S (2021). Epidemiology and associated factors of polypharmacy in older patients in primary care: a northern Italian cross-sectional study. BMC Geriatr.

[32] American Geriatrics Society Beers Criteria Update Expert P (2012). American Geriatrics Society updated beers criteria for potentially inappropriate medication use in older adults. J Am Geriatr Soc.

[33] Emilia-Romagna SSR (2014). Appropriatezza prescrittiva nella popolazione anziana - Elenco farmaci potenzialmente inappropriati ed eventuali alternative terapeutiche.

[34] UpToDate® Drugs & Drug Interaction Database. 2016. https://www.uptodate.com/home/uptodate-drug-interactions-tool. Accessed 06 Mar 2020.

[35] Herdman M, Gudex C, Lloyd A, Janssen M, Kind P, Parkin D (2011). Development and preliminary testing of the new five-level version of EQ-5D (EQ-5D-5L). Qual Life Res.

[36] Rinaldi P, Mecocci P, Benedetti C, Ercolani S, Bregnocchi M, Menculini G (2003). Validation of the five-item geriatric depression scale in elderly subjects in three different settings. J Am Geriatr Soc.

[37] Brooke P, Bullock R (1999). Validation of a 6 item cognitive impairment test with a view to primary care usage. Int J Geriatr Psychiatry.

[38] Peters TJ, Richards SH, Bankhead CR, Ades AE, Sterne JA (2003). Comparison of methods for analysing cluster randomized trials: an example involving a factorial design. Int J Epidemiol.

[39] EuroQoL. EQ-5D-5L Valuation Crosswalk Index Value Calculator. https://euroqol.org/eq-5d-instruments/eq-5d-5l-about/valuation-standard-value-sets/crosswalk-index-value-calculator/. Accessed 22 Sept 2014.

[40] Szende A, Janssen B, Cabases J (2014). Self-Reported Population Health: An International Perspective based on EQ-5D. https://eq-5dpublications.euroqol.org/download?id=0_54006&fileId=54415. Accessed 02 April 2019. 29787044

[41] EuroQol Research Foundation. EQ-5D-5L User Guide. 2019. file:///C:/Users/AM-MG/Downloads/EQ-5D-5L-English-User-Guide_version-3.0-Sept-2019-secured.pdf. Accessed 10 June 2020.

[42] Rieckert A, Reeves D, Altiner A, Drewelow E, Esmail A, Flamm M (2020). Use of an electronic decision support tool to reduce polypharmacy in elderly people with chronic diseases: cluster randomised controlled trial. BMJ..

[43] Sheppard JP, Burt J, Lown M, Temple E, Lowe R, Fraser R (2020). Effect of antihypertensive medication reduction vs usual care on short-term blood pressure control in patients with hypertension aged 80 years and older: the OPTIMISE randomized clinical trial. JAMA..

[44] Zermansky AG, Alldred DP, Petty DR, Raynor DK, Freemantle N, Eastaugh J (2006). Clinical medication review by a pharmacist of elderly people living in care homes--randomised controlled trial. Age Ageing.

[45] Tamura BK, Bell CL, Lubimir K, Iwasaki WN, Ziegler LA, Masaki KH (2011). Physician intervention for medication reduction in a nursing home: the polypharmacy outcomes project. J Am Med Dir Assoc.

[46] Williams ME, Pulliam CC, Hunter R, Johnson TM, Owens JE, Kincaid J (2004). The short-term effect of interdisciplinary medication review on function and cost in ambulatory elderly people. J Am Geriatr Soc.

[47] Gnjidic D, Le Couteur DG, Kouladjian L, Hilmer SN (2012). Deprescribing trials: methods to reduce polypharmacy and the impact on prescribing and clinical outcomes. Clin Geriatr Med.

[48] Stuhec M, Flegar I, Zelko E, Kovacic A, Zabavnik V. Clinical pharmacist interventions in cardiovascular disease pharmacotherapy in elderly patients on excessive polypharmacy : a retrospective pre-post observational multicentric study. Wien Klin Wochenschr. 2021. 10.1007/s00508-020-01801-y 33471149

[49] Rankin A, Cadogan CA, Patterson SM, Kerse N, Cardwell CR, Bradley MC (2018). Interventions to improve the appropriate use of polypharmacy for older people. Cochrane Database Syst Rev.

[50] Johansson T, Abuzahra ME, Keller S, Mann E, Faller B, Sommerauer C (2016). Impact of strategies to reduce polypharmacy on clinically relevant endpoints: a systematic review and meta-analysis. Br J Clin Pharmacol.

[51] Page AT, Clifford RM, Potter K, Schwartz D, Etherton-Beer CD (2016). The feasibility and effect of deprescribing in older adults on mortality and health: a systematic review and meta-analysis. Br J Clin Pharmacol.

[52] Howard RL, Avery AJ, Slavenburg S, Royal S, Pipe G, Lucassen P (2007). Which drugs cause preventable admissions to hospital? A systematic review. Br J Clin Pharmacol.

[53] Garfinkel D, Mangin D (2010). Feasibility study of a systematic approach for discontinuation of multiple medications in older adults: addressing polypharmacy. Arch Intern Med.

[54] Mahlknecht A, Abuzahra ME, Piccoliori G, Enthaler N, Engl A, Sonnichsen A (2016). Improving quality of care in general practices by self-audit, benchmarking and quality circles. Wien Klin Wochenschr.

[55] Furthauer J, Flamm M, Sonnichsen A (2013). Patient and physician related factors of adherence to evidence based guidelines in diabetes mellitus type 2, cardiovascular disease and prevention: a cross sectional study. BMC Fam Pract.

[56] Doherty AJ, Boland P, Reed J, Clegg AJ, Stephani AM, Williams NH, et al. Barriers and facilitators to deprescribing in primary care: a systematic review. BJGP Open. 2020;4(3). 10.3399/bjgpopen20X101096 PMC7465575 32723784

[57] Rozsnyai Z, Jungo KT, Reeve E, Poortvliet RKE, Rodondi N, Gussekloo J (2020). What do older adults with multimorbidity and polypharmacy think about deprescribing? The LESS study - a primary care-based survey. BMC Geriatr.

[58] Whitman A, DeGregory K, Morris A, Mohile S, Ramsdale E (2018). Pharmacist-led medication assessment and deprescribing intervention for older adults with cancer and polypharmacy: a pilot study. Support Care Cancer.

[59] Anthierens S, Tansens A, Petrovic M, Christiaens T (2010). Qualitative insights into general practitioners views on polypharmacy. BMC Fam Pract.

[60] Farrell B, Pottie K, Thompson W, Boghossian T, Pizzola L, Rashid FJ (2017). Deprescribing proton pump inhibitors: evidence-based clinical practice guideline. Can Fam Physician.

[61] AMDA - the Society for Post-acute and Long-term Care Medicine. Five things physicians and patients should question. Choosing Wisely website. 2013. Available from: http://www.choosingwisely.org/doctor-patient-lists/amda/. .

[62] Morin L, Johnell K, Laroche ML, Fastbom J, Wastesson JW (2018). The epidemiology of polypharmacy in older adults: register-based prospective cohort study. Clin Epidemiol.

[63] Sarzynski EM, Luz CC, Rios-Bedoya CF, Zhou S (2014). Considerations for using the 'brown bag' strategy to reconcile medications during routine outpatient office visits. Qual Prim Care.

