# Supplementary material for: Expert-based medication reviews to reduce polypharmacy in older patients in primary care: a northern-Italian cluster-randomised controlled trial
Source: BMC Geriatr. 2021 Nov 23;21:659. doi: 10.1186/s12877-021-02612-0 (PMC8609829; doi:10.1186/s12877-021-02612-0)
Supplement: Supplementary file 1 — Additional file 1:. The additional file contains the Supplementary Tables I-IV (word format), which report additional results information: Biometric and laboratory values at T0, list and frequency of symptoms at T0 and T2. Most common Beers-listed drug classes, drug-drug interactions (DDIs), and drug classes involved in DDIs at T0. Descriptive within group-analysis (longitudinal analysis T0 – T2) for the intervention group and the control group. Depiction of the case report form used for collection of data. [file 12877_2021_2612_MOESM1_ESM.docx]

# Supplementary tables

### Supplementary Tab.I: Laboratory values and biometrical parameters at baseline (T_0_), symptoms at T_0_/T_2_

| **Biometric and laboratory parameters (T_0_)** | | | **Intervention group** (n=307) | **Control group** (n=272) | **p-value** |
| --- | --- | --- | --- | --- | --- |
|  | **Unit** | **n** | **Median (IQR)** | **Median (IQR)** |  |
| Weight | kg | 579 | 72.0 (62.0 – 81.0) | 71.5 (62.0 – 80.0) | 0.729 ^i^ |
| Height | cm | 579 | 164 (158 – 170) | 163.5 (158 – 170) | 0.577 ^i^ |
| Systolic blood pressure | mmHg | 573 | 135 (125 – 140) | 135 (130 – 145) | 0.540 ^i^ |
| Diastolic blood pressure | mmHg | 572 | 80 (70 – 80) | 75 (70 – 80) | 0.215 ^i^ |
| Cardiac frequency | beats/min | 491 | 70 (64 – 79) | 70 (65 – 79) | 0.494 ^i^ |
| Glycosylated haemoglobin (HbA1c) | % | 245 | 6.8 (6.3 – 7.6) | 6.7 (6.2 – 7.2) | 0.239 ^i^ |
| Haemoglobin | g/dl | 278 | 13.0 (11.7 – 13.9) | 13.1 (12.1 – 14.1) | 0.098 ^i^ |
| Glomerular filtration rate (GFR) | ml/min | 350 | 57.3 (45.1 – 72.5) | 59.2 (43.9 – 72.7) | 0.591 ^i^ |
| Glutamate-pyruvate-transaminase (GPT) | U/l | 252 | 16.0 (11.3 – 24.0) | 17.0 (13.0 – 22.8) | 0.535 ^i^ |
| Gamma-glutamyltransferase (γ-GT) | U/l | 258 | 19.0 (12.0 – 28.0) | 18.0 (13.0 – 26.0) | 0.986 ^i^ |
| Potassium | mmol/l | 285 | 4.3 (4.0 – 4.7) | 4.4 (4.1 – 4.7) | 0.141 ^i^ |
| Sedimentation rate | mm/h | 187 | 25.0 (16.0 – 37.0) | 20.0 (11.5 – 34.5) | 0.142 ^i^ |
| Brain natriuretic peptide (BNP/NT-proBNP) | ng/l | 78 | 659 (408 – 1336) | 853 (439 – 2225) | 0.267 ^i^ |
| International normalised ration (INR) |  | 74 | 2.5 (1.8 – 3.2) | 2.4 (2.1 – 3.0) | 0.614 ^i^ |
| **Frequency of symptoms (T_0_)** | | | **Intervention group** (n=307) | **Control group** (n=272) | **p-value** |
| **12 months before T_0_** | | | | | |
| Cardiovascular problems (%) | | | 61 (19.9%) | 27 (9.9%) | **0.001** ^ii^ |
| Anaemia (%) | | | 18 (5.9%) | 3 (1.1%) | **0.003** ^ii^ |
| Gastrointestinal bleeding (%) | | | 1 (0.3%) | 2 (0.7%) | 0.603 ^ii^ |
| **1 month before T_0_** | | | | | |
| Nausea (%) | | | 27 (8.8%) | 8 (2.9%) | **0.005** ^ii^ |
| Vertigo (%) | | | 95 (30.9%) | 50 (18.4%) | **0.001** ^ii^ |
| Pain (%) | | | 105 (34.2%) | 65 (23.9%) | **0.008** ^ii^ |
| Obstipation (%) | | | 42 (13.7%) | 28 (10.3%) | 0.251 ^ii^ |
| Diarrhoea (%) | | | 10 (3.3%) | 10 (3.7%) | 0.823 ^ii^ |
| Dyspnoea (%) | | | 54 (17.6%) | 23 (8.5%) | **0.001** ^ii^ |
| Angina pectoris (%) | | | 15 (4.9%) | 6 (2.2%) | 0.118 ^ii^ |
| Weight loss >2kg (%) | | | 10 (3.3%) | 3 (1.1%) | 0.096 ^ii^ |
| **Frequency of symptoms (T_2_)** | | | **Intervention group** (n=250) | **Control group** (n=233) | **p-value** |
| **12 months before T_2_** | | | | | |
| Cardiovascular problems (%) | | | 26 (10.4%) | 18 (7.7%) | 0.344 ^ii^ |
| Anaemia (%) | | | 12 (4.8%) | 9 (3.9%) | 0.661 ^ii^ |
| Gastrointestinal bleeding (%) | | | 0 (0%) | 2 (0.9%) | 0.233 ^ii^ |
| **1 month before T_2_** | | | | | |
| Nausea (%) | | | 16 (6.4%) | 41 (17.6%) | **<0.001**^ii^ |
| Vertigo (%) | | | 84 (33.6%) | 58 (24.9%) | **0.037** ^ii^ |
| Pain (%) | | | 111 (44.4%) | 110 (47.2%) | 0.584 ^ii^ |
| Obstipation (%) | | | 39 (15.6%) | 55 (23.6%) | **0.029** ^ii^ |
| Diarrhoea (%) | | | 15 (6.0%) | 28 (12.0%) | **0.025** ^ii^ |
| Dyspnoea (%) | | | 37 (14.8%) | 23 (9.9%) | 0.128 ^ii^ |
| Angina pectoris (%) | | | 4 (1.6%) | 0 (0%) | 0.124 ^ii^ |
| Weight loss >2kg (%) | | | 5 (2.0%) | 2 (0.9%) | 0.452 ^ii^ |
| *Total number of symptoms (1 month before T_2_)* | | | *311* | *317* | - |
| *Median per patient (IQR)* | | | *1 (0 – 2)* | *1 (0 – 2)* | *0.143* *^i^* |

^i^ Mann-Whitney-U test, ^ii^ Fishers exact test

*IQR* Interquartile quartile range

### Supplementary Tab.II: Common Beers-listed drug classes, drug-drug interactions (DDIs), and drug classes involved in DDIs

| **Pre-review of drug regimens at T_0_ (before the intervention)** | **Intervention group** n=307 | **Control group** n=272 |
| --- | --- | --- |
| **Beers-listed drugs** | **n drugs (% of patients)** | **n drugs (% of patients)** |
| Benzodiazepines, Zolpidem | 49 (16.0%) | 65 (23.9%) |
| NSAIDs, COX-2-inhibitors (Coxibe) | 25 (8.1%) | 13 (4.8%) |
| Alpha-blockers | 23 (7.5%) | 7 (2.6%) |
| Antiarrhythmics | 22 (7.2%) | 15 (5.5%) |
| CCBs | 14 (4.6%) | 12 (4.4%) |
| Diuretics incl. Spironolacton | 12 (3.9%) | 17 (6.3%) |
| Antidepressants and antipsychotics | 12 (3.9%) | 8 (2.9%) |
| Antithrombotic drugs, mainly Ticlopidine | 9 (2.9%) | 13(4.8%) |
| Cardiac glycosides | 4 (1.3%) | 9 (3.3%) |
| Others (e.g. Tiotropium, Pioglitazone, Phenobarbital, Tramadol) | 11 (3.6%) | 1 (0.37%) |
| *Total number of Beers-listed drugs* | *181* | *160* |
| **Drug-drug interactions ^§^** | **n DDIs (% of patients)** | **n DDIs(% of patients)** |
| Acenocoumarol/Warfarin + Allopurinol | 16 (5.2%) | 16 (5.9%) |
| Alendronate + Calcium carbonate | 16 (5.2%) | 19 (7.0%) |
| Amlodipine/Lercanidipine + Simvastatin | 16 (5.2%) | 11 (4.0%) |
| Levothyroxine + Calcium carbonate | 14 (4.6%) | 12 (4.4%) |
| Allopurinol + Ramipril/Lisinopril/Enalapril | 15 (4.9%) | 6 (2.2%) |
| Bisoprolol + Tamsulosin | 8 (2.6%) | 10 (3.7%) |
| Acenocoumarol/Warfarin + Acetylsalicylic acid | 6 (2.0%) | 10 (3.7%) |
| Ibuprofen/Diclofenac + Acetylsalicylic acid | 10 (3.3%) | 5 (1.8%) |
| Acetylsalicylic acid + Etoricoxib | 2 (0.65%) | 10 (3.7%) |
| Clopidogrel + Pantoprazole | 9 (2.9%) | 2 (0.7%) |
| Bisoprolol + Doxazosin | 6 (2.0%) | 3 (1.1%) |
| Ibuprofen + Warfarin | 6 (2.0%) | 3 (1.1%) |
| Ibuprofen + Furosemide | 4 (1.3%) | 4 (1.5%) |
| Prednison + Calcium carbonate | 3 (0.97%) | 4 (1.5%) |
| *Total number of DDIs* | *380* | *396* |
| **Drug classes involved in D ^§^ or X ^§^ DDIs** | **n drugs (% of patients)** | **n drugs (% of patients)** |
| Antithrombotic / anticoagulant drugs | 87 (28.3%) | 89 (32.7%) |
| Antidepressants, antipsychotics | 69 (22.5%) | 65 (23.9%) |
| ARBs, ACE-inhibitors | 46 (15.0%) | 15 (5.5%) |
| Calcium carbonate | 44 (14.3%) | 33 (12.1%) |
| Beta-blockers | 43 (14.0%) | 38 (14.0%) |
| Alpha-blockers | 42 (13.7%) | 30 (11.0%) |
| Statins | 38 (12.4%) | 26 (9.6%) |
| CCBs | 31 (10.1%) | 23 (8.5%) |
| Drugs for gout treatment-Allopurinol | 28 (9.1%) | 20 (7.4%) |
| NSAIDs, COX-2-inhibitors (Coxibe) | 21 (6.8%) | 41 (15.1%) |

^§^ Drug-drug interactions: category D = consider drug modification, category X = avoid combination (1)

*n* Number of drugs, *NSAIDs* Non-steroidal anti-inflammatory drugs, *COX* Cyclooxygenase, *CCBs* Calcium channel blockers, *DDIs* Drug-drug interactions, *ARBs* Angiotensin II receptor antagonists, *ACE* Angiotensin converting enzyme

### Supplementary Tab.III: Descriptive within group-analysis (T_0_-T_2_) for both treatment groups

| **Outcomes** ^§^ | **Intervention group** | | **Control group** | |
| --- | --- | --- | --- | --- |
|  | **T_0_** | **T_2_** | **T_0_** | **T_2_** |
| **Non-elective hospital admissions** | **n = 307** | **n = 281** | **n = 272** | **n = 257** |
| Patients with ≥1 hospital admission (%) | 85 (27.7%) ^§§^ | 103 (36.7%) | 37 (13.6%) ^§§^ | 68 (26.5%) |
| Total number of hospital admissions | 85 ^§§^ | 122 | 37 ^§§^ | 74 |
| Median (IQR) | 0 (0 – 1) ^§§^ | 0 (0 – 1) | 0 (0 – 0) ^§§^ | 0 (0 – 0.8) |
| **Falls** | **n = 307** | **n = 281** | **n = 272** | **n = 257** |
| Patients with ≥1 falls (%) | 66 (21.5%) ^§§^ | 58 (20.6%) | 28 (10.3%) ^§§^ | 73 (28.4%) |
| Total number of falls | 66 ^§§^ | 65 | 28 ^§§^ | 76 |
| Median (IQR) | 0 (0 – 0) ^§§^ | 0 (0 – 0) | 0 (0 – 0) ^§§^ | 0 (0 – 1) |
| **Fractures** | **n = 307** | **n = 281** | **n = 272** | **n = 257** |
| Patients with ≥1 fractures (%) | 23 (7.5%) ^§§^ | 25 (8.9%) | 11 (4.0%) ^§§^ | 13 (5.1%) |
| Total number of fractures | 23 ^§§^ | 26 | 11 ^§§^ | 13 |
| Median (IQR) | 0 (0 – 0) ^§§^ | 0 (0 – 0) | 0 (0 – 0) ^§§^ | 0 (0 – 0) |
| **Number of drug prescriptions** | **n = 307** | **n = 250** | **n = 272** | **n = 235** |
| Total number of drugs | 2,914 | 2,218 | 2,700 | 2,140 |
| Median number of drugs (IQR) | 9 (8 – 10) | 8 (7 – 10) | 9 (8 – 11) | 9 (8 – 10) |
| **EQ-5D-5L index** | **n = 307** | **n = 250** | **n = 272** | **n = 235** |
| Median (IQR) | 0.815  (0.710 – 0.910) | 0.806  (0.698 – 0.909) | 0.810  (0.716 – 0.909) | 0.806  (0.701 – 0.909) |
| **EQ-VAS score** | **n = 307** | **n = 250** | **n = 272** | **n = 234** |
| Median (IQR) | 60 (50 – 80) | 60 (50 – 76.3) | 65 (50 – 78.8) | 60 (50 – 70) |
| **5-GDS:** Score ≥ 2 points (affective impairment) | **n = 307** | **n = 250** | **n = 272** | **n = 235** |
| n patients (%) | 94 (30.6%) | 105 (42.0%) | 76 (27.9%) | 81 (34.5%) |
| **6-CIT:** Score ≥ 8 points (cognitive impairment) | **n = 307** | **n = 250** | **n = 272** | **n = 235** |
| n patients (%) | 77 (25.1%) | 64 (25.6%) | 81 (29.8%) | 61 (26.0%) |

^§^ Within group-analysis was conducted for all outcomes from which parameters were collected at baseline and at T_2_ (secondary endpoints except mortality which was assessed exclusively at T_2_)

^§§^ Within 12 months before T_0_

*IQR* Interquartile range, *EQ-5D* 5-Item questionnaire measuring health-related quality of life, *VAS* Visual analogue scale, *5-GDS* 5-Item Geriatric Depression Scale, *6-CIT* 6-Item Cognitive Impairment Test

### Supplementary Tab.IV: Case report form used for collection of data in the PRIMA study

| 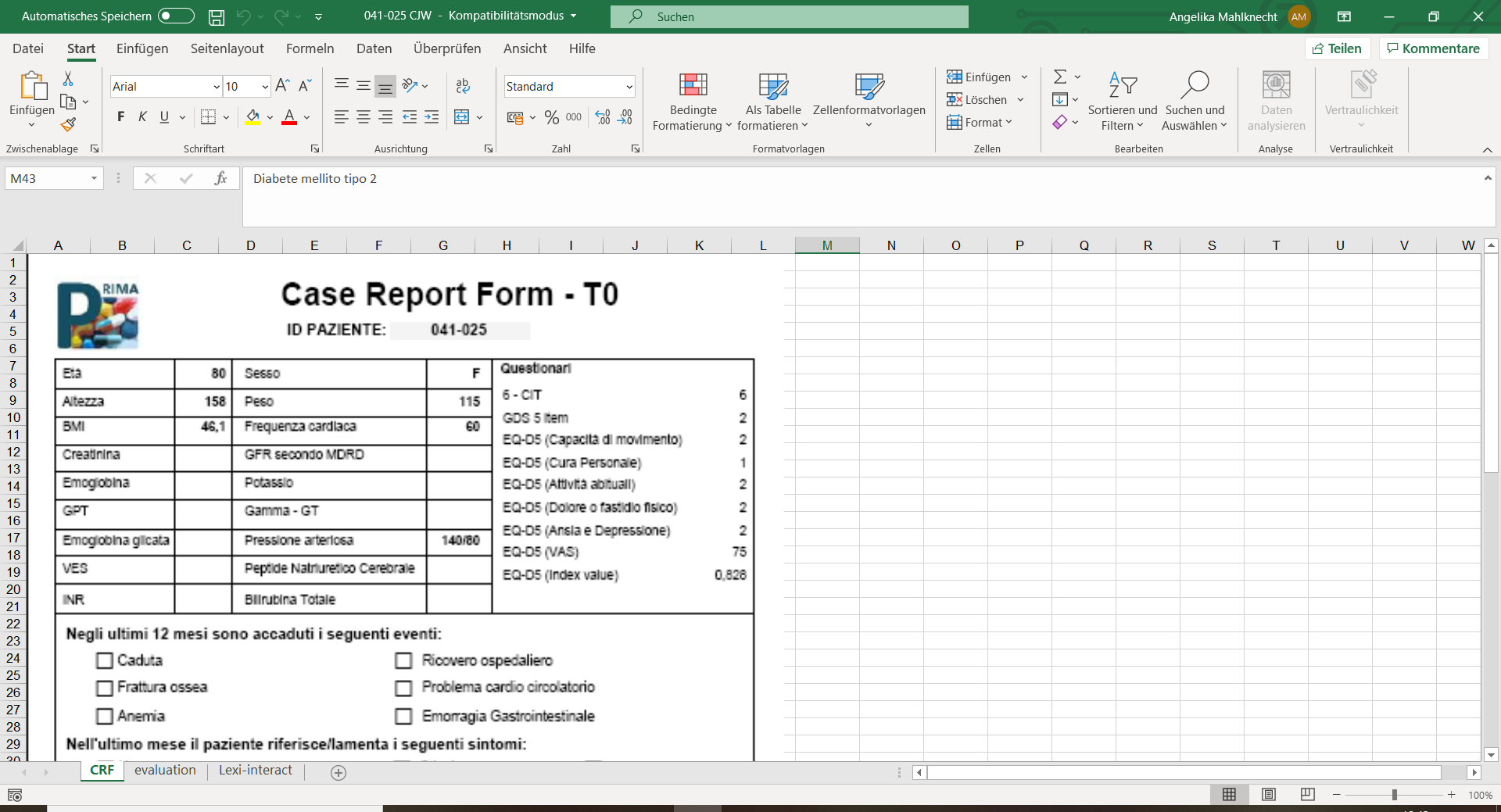 | | **Case Report Form – T_0_**  Patient ID: ___________________________ | | | | | | | |
| --- | --- | --- | --- | --- | --- | --- | --- | --- | --- |
| **Biometric and laboratory parameters** | | | | | | | **Questionnaires** | | |
| Age |  | | Sex | | |  | 5-GDS score | |  |
| Height |  | | Weight | | |  | 6-CIT score | |  |
| BMI |  | | Cardiac frequency | | |  | EQ-5D Mobility | |  |
| Creatinine |  | | Glomerular filtration rate | | |  | EQ-5D Selfcare | |  |
| Haemoglobin |  | | Potassium | | |  | EQ-5D Usual activities | |  |
| GPT |  | | γ-GT | | |  | EQ-5D Pain / discomfort | |  |
| HbA1c |  | | Blood pressure | | |  | EQ-5D Anxiety / depression | |  |
| Sedimentation rate |  | | BNP/NT-proBNP | | |  | EQ-5D Index value | |  |
| INR |  | |  | | |  | EQ-VAS score | |  |
| **Occurred events within the last 12 months:** | | | | | | | | | |
| Fall  Fracture  Anaemia | | | | | Hospital admission  Cardiovascular problems  Gastrointestinal bleeding | | | | |
| **Occurred events within the last month:** | | | | | | | | | |
| Nausea  Vertigo  Pain  Obstipation | | | | | Diarrhoea  Dyspnoea  Angina pectoris  Weight loss >2kg | | | | |
| **Current medication** | | | **ATC** | **Dosage** | | **Frequency per die** | | **Units of administration** | |
|  | | |  |  | |  | |  | |
|  | | |  |  | |  | |  | |
|  | | |  |  | |  | |  | |
|  | | |  |  | |  | |  | |
|  | | |  |  | |  | |  | |
|  | | |  |  | |  | |  | |
|  | | |  |  | |  | |  | |
|  | | |  |  | |  | |  | |
|  | | |  |  | |  | |  | |
| **Current diagnoses** | | | | **ICD-10** | | **Date of registration** | | | |
|  | | | |  | |  | | | |
|  | | | |  | |  | | | |
|  | | | |  | |  | | | |
|  | | | |  | |  | | | |
|  | | | |  | |  | | | |
|  | | | |  | |  | | | |
|  | | | |  | |  | | | |
|  | | | |  | |  | | | |
| *Date of CRF: ____________________________________* | | | | | | | | | |

*BMI* Body mass index, *GPT* Glutamate-pyruvate-transaminase, *γ-GT* Gamma-glutamyltransferase, *HbA1c* Glycosylated haemoglobin, *BNP/NT-proBNP* Brain natriuretic peptide, *INR* International normalised ratio, *5-GDS* 5-Item Geriatric Depression Scale, *6-CIT* 6-Item Cognitive Impairment Test, *EQ-5D* 5-Item questionnaire measuring health-related quality of life, *VAS* Visual analogue scale, *ATC* Anatomical Therapeutic Chemical classification, *ICD-10* International Classification of Diseases, revision 10, *CRF* Case report form
